# Morphology-based taxonomic re-assessment of the Arctic lamprey, *Lethenteron camtschaticum* (Tilesius, 1811) and taxonomic position of other members of the genus

**DOI:** 10.3897/zookeys.991.54938

**Published:** 2020-11-11

**Authors:** Alexander M. Naseka, Claude B. Renaud

**Affiliations:** 1 Naturhistorisches Museum Wien, Burgring 7, 1010 Vienna, Austria Naturhistorisches Museum Wien Vienna Austria; 2 Centre for Arctic Knowledge and Exploration, Research & Collections, Canadian Museum of Nature, P.O. Box 3443, Station D, Ottawa, ON K1P 6P4 Canada Canadian Museum of Nature Ottawa Canada

**Keywords:** Arctic lamprey, *Lethenteron* species, synonyms, taxonomic key

## Abstract

The lamprey genus *Lethenteron* Creaser & Hubbs, 1922 is widespread across Eurasia and North America, but the number and distribution of its constituent species is not firmly established. After a morphological examination of extant type material of the currently recognized species and their synonyms, *Lethenteron mitsukurii* (Hatta, 1901) is resurrected with *Le. matsubarai* Vladykov & Kott, 1978 as its junior synonym. Amongst nonparasitic species *Le. reissneri* (Dybowski, 1869) and *Le. mitsukurii* are confirmed as present in Japan and the former is also present on Sakhalin. An in-depth study of large samples of nonparasitic lamprey adults from Japan and Sakhalin Island is needed to determine whether the lower trunk myomere (< 66) individuals from these areas represent one or more undescribed species, or *Le. mitsukurii*, or *Le. reissneri*, or a mixture of these three alternatives. The material from the Anadyr Estuary identified by Berg (1931, 1948) as *Lampetra japonica kessleri* has been re-identified as *Le. camtschaticum* and there is no evidence that *Le. kessleri* occurs there. *Lethenteron reissneri* is reported from the Angara River system, Yenisei River drainage, Russia. *Lethenteron alaskense* Vladykov & Kott, 1978 is provisionally considered to be a junior synonym of *Le. kessleri* (Anikin, 1905). *Petromyzon ernstii* Dybowski, 1872, *Ammocoetes aureus* Bean, 1881, *Petromyzon dentex* Anikin, 1905, *Lampetra mitsukurii major* Hatta, 1911, and *Lampetra japonica septentrionalis* Berg, 1931 are junior synonyms of *Petromyzon marinus camtschaticus* Tilesius, 1811. A key is provided to adults of the six species recognized as belonging in the genus *Lethenteron*.

## Introduction

The lamprey genus *Lethenteron* Creaser & Hubbs, 1922 is widely distributed across the Northern Hemisphere, but the number and distribution of its constituent species is not firmly established. Originally erected by Creaser and Hubbs (1922) as a subgenus of *Entosphenus* Gill, 1862, the cladistic study of Gill et al. (2003) based on morphological characters of parasitic species only, showed that *Entosphenus* is a monophyletic group supported by two synapomorphies, and *Lethenteron* belongs to a sister clade, also supported by two synapomorphies, that includes *Eudontomyzon* Regan, 1911 and *Lampetra* Bonnaterre, 1788. Furthermore, the cladogram by Gill et al. (2003) suggests that *Lethenteron* is sister to *Eudontomyzon*-*Lampetra*. However, no derived character defined *Lethenteron*, while the latter clade was supported by a single synapomorphy. Until a phylogenetic study more definitely resolves the relationships among the three genera, we choose to recognize *Lethenteron* as a distinct genus. According to Vladykov and Follett (1967), the genus *Lethenteron* is characterized by the presence of a single row of posterial teeth, a 2–2–2 endolateral formula, and the absence of exolateral teeth. However, the latter character shows variation (Kott 1974; Vladykov and Kott 1978a; Renaud and Naseka 2015).

Kottelat (1997) resurrected *Lethenteron camtschaticum* (Tilesius, 1811) for the Arctic lamprey, and thus, the long-standing name *Lethenteron japonicum* (von Martens, 1868) became the junior synonym. The type for *Le. camtschaticum* being lost, Kottelat (1997) designated the lectotype of *Le. japonicum* (ZMB 6475) as the neotype for the former making the type locality for *Le. camtschaticum* Tokyo (appeared as Jeddo) and Yokohama, Honshu Island, Japan as defined by the neotype. However, while the scientific name for the taxon has been fixed, its taxonomic limits are still not clear. Berg (1931) separated what he called *Lampetra japonica* into three subspecies: the nominotypical subspecies, to which he gave the common name Pacific river lamprey, distributed in the North Pacific Ocean basin in an arc from the Sea of Japan basin (Korean Peninsula, areas of Gensan, now known as Wŏnsan in North Korea and Fusan, now known as Busan in South Korea; Japan; Russian Far East, Tumen River and Suchan River, now known as Partizanskaya River), the Amur River, the Kamchatka Peninsula and across to the Yukon River in North America; a second subspecies *La. j. septentrionalis* Berg, 1931, to which he gave the common name Arctic lamprey, restricted to the Arctic Ocean basin from the White Sea basin to the Ob’ River drainage; and a third subspecies *La. j. kessleri* (Anikin, 1905), the Siberian lamprey, distributed in the intervening zone between the two (Ob’ River to Kolyma and Anadyr rivers, and Sakhalin Island).

Berg (1931) considered *La. j. japonica* and *La. j. septentrionalis* to be large migratory (anadromous) forms in which the larvae at metamorphosis were much smaller than the adults and *La. j. kessleri* to be a small non-migratory (i.e., resident) form in which the larvae at metamorphosis were larger than the adults. Altukhov et al. (1958) reported that anadromous *La. japonica* migrate 400 or more kilometers up the Severnaya (or Northern) Dvina and Mezen’ rivers, White Sea basin, while in the Amur River Morozova (1956) reported upstream migrations varying between 766 and 905 km, and according to Chereshnev (2008), even up to a remarkable 1,700 km. Although Berg (1931) made no statement in regard to adult feeding, the implication of his dichotomy was that the first two were parasitic lampreys while the third was a nonparasitic lamprey. Furthermore, Berg (1931) likened the relationship between *La. j. septentrionalis* and *La. j. kessleri* to that of *La. fluviatilis* (Linnaeus, 1758) and *La. planeri* (Bloch, 1784), thus corroborating the parasitic-nonparasitic argument. Another level of complexity introduced by Berg (1931) is the concept that migratory lampreys may be represented by two sympatric forms; one large and one small (i.e., f. praecox), and that the small, earlier-maturing form may be migratory or paradoxically resident (i.e., non-migratory) in lakes. A single female of *La. j. septentrionalis* 247 mm total length from the Onega River (type locality), Russia was identified by Berg (1931) as belonging to the forma praecox. However, *praecox* is not an available name according to article 1.3 of the International Code of Zoological Nomenclature (ICZN 1999) because it was proposed as an infrasubspecific rank. Berg (1948), without justifying his action, synonymized *La. j. septentrionalis* with the nominotypical subspecies under the common name Arctic lamprey, and increased the re-defined taxon’s western range to include the Barents Sea basin at least to Motovsky Bay, Russia and perhaps to Varanger Fjord, Norway, and in the eastern range included mainland rivers of the Okhotsk Sea basin (Okhota, Kukhtui, Taui, and Ola) and Sakhalin Island, Russia. Despite this, the common name Pacific lamprey has persisted in the Russian literature in reference to this taxon (Birman 1950; Morozova 1956; Nikol’sky 1956; Martynov 2002; Gritsenko et al. 2006; Bugayev et al. 2007; Savvaitova et al. 2007; Chereshnev 2008).

Berg (1931, 1948) noted the wide discontinuity in the distribution of the anadromous populations of *La. j. japonica*; none being present between the Gulf of Ob’ and Kamchatka. However, Berg (1948) still recognized the subspecies *La. j. kessleri* and increased its western range to include the Pechora River, Barents Sea basin and suggested that this taxon was probably also present in Alaska. Ioganzen (1935a, 1935b) compared the morphometrics and dentition of *La. j. kessleri* and *La. j. septentrionalis* collected sympatrically in the Ob’ River drainage including at the type locality of the former (Tom’ River near Tomsk), and, other than total length (i.e., 132–207 mm versus 215–408 mm, respectively), could not find any significant differences between the two. Furthermore, Ioganzen (1935a) dismissed the wide discontinuity between the distributions of *La. j. septentrionalis* and *La. j. japonica* by suggesting that their similar dentition was not indicative of their common origin and that *La. j. septentrionalis* was in fact derived from *La. fluviatilis*, and should therefore be called *La. fluviatilis septentrionalis*. However, Ioganzen (1935b) accepted the close phylogenetic relationship between *La. j. kessleri* and its presumed ancestor *La. j. japonica*.

Holčík (1986a, 1986b) recognized the genus *Lethenteron* and elevated *Le. kessleri* to the rank of species, as originally proposed by Anikin (1905) followed by Poltorykhina (1974). Holčík (1986b) stated that *Le. kessleri* was most probably nonparasitic and usually indistinguishable morphologically from other satellite (i.e., nonparasitic) species of *Le. camtschaticum* (appeared as *Le. japonicum*), namely, *Le. reissneri* (Dybowski, 1869), *Le. wilderi* (Gage in Jordan & Evermann, 1896), *Le. meridionale* Vladykov, Kott & Pharand-Coad, 1975, *Le. alaskense* Vladykov & Kott, 1978, and *Le. matsubarai* Vladykov & Kott, 1978, and that these are possibly conspecific. He thus called for an urgent critical revision, earlier advocated by Hubbs and Potter (1971), and this has been most recently reiterated by Dyldin et al. (2019a). Two of the species recognized by Holčík (1986b), *Le. wilderi* and *Le. meridionale*, are junior synonyms of different species. The former is a questionable synonym of *Lethenteron appendix* (DeKay, 1842) (see Renaud 2011) and the latter a synonym of *Lampetra aepyptera* (Abbott, 1860) (see Walsh and Burr 1981). The generic allocation of *La. aepyptera* is not clear. It is either *Lampetra* or *Okkelbergia* Creaser & Hubbs, 1922, but not *Lethenteron* (see Potter et al. 2015), and therefore, it will not be dealt with further.

Ren et al. (2016) examined the mitogenome of *Le. camtschaticum*, *Le. reissneri*, *Le. appendix*, *Le. morii*, *La. aepyptera*, and *La. fluviatilis*. Their phylogenetic tree using a maximum likelihood method with the Tamura-Nei substitution model suggested that there were two *Lethenteron* lineages; one consisting of the clade *Le. camtschaticum*-*Le. appendix*-*Le. morii*-*Le. reissneri* sister to a *La. aepyptera*-*La. fluviatilis* clade and another consisting of *Le. camtschaticum*-*Le. reissneri* that was sister to those two clades. Ren et al. (2016) stated that morphological comparisons of closely related lampreys can be difficult, and may have resulted in mistaken species identification leading to this confusing result. Misidentifications notwithstanding, it is difficult to explain how species of *Lampetra* distributed in eastern North America (*La. aepyptera*) and Europe (*La. fluviatilis*) are more closely related to Asian species of *Lethenteron* (*Le. camtschaticum* and *Le. reissneri*), but not to another species pair of *Le. camtschaticum*-*Le. reissneri*, unless the latter represent entirely new species. Moreover, the resolution of the relationships between *Lampetra*, *Lethenteron*, and *Eudontomyzon* is beyond the scope of this study and will be the object of future inquiry.

The goals of this study are to:

- re-assess the taxonomic status of the three subspecies of Lampetra japonica proposed by Berg (1931), as well as that of the following nominal taxa that have been considered as synonyms, either tentatively or not, of one or the other of these putative subspecies by Berg (1931, 1948): Petromyzon ernstii Dybowski, 1872 described from the mouth of the Amur River, Russia, Ammocoetes aureus Bean, 1881 described from Anvik, Yukon River, Alaska, U.S.A., and Petromyzon dentex Anikin, 1905 described from the mouth of the Yenisei River, near Gol’chikha, Russia;
- establish the relationship with Le. camtschaticum of two lamprey species, Lampetra mitsukurii Hatta, 1901 described from small watercourses on Hondo (= Honshu), Shikoku, Kyushu, and Hokkaido islands, Japan and Le. matsubarai described from Shokotsu River, Hokkaido Island, Japan. The first was synonymized with La. reissneri by Berg (1931) and later tentatively resurrected by Hubbs and Potter (1971), while the second was synonymized with Le. kessleri by Iwata et al. (1985).
- re-evaluate synonymization of Le. kessleri with Le. reissneri by Yamazaki et al. (2006) and the two names with Le. camtschaticum by Kucheryavyy (2014) as not based on a re-examination of all of the relevant type material.
- establish relationships of Lampetra mitsukurii minor Hatta, 1911 and La. m. major Hatta, 1911 with Le. camtschaticum. Berg (1931) considered La. m. minor a synonym of La. reissneri and La. m. major a synonym of La. japonica japonica.

Molecular data of Lang et al. (2009), Pu et al. (2016), and Ren et al. (2016) have suggested that Lampetra (Eudontomyzon) morii Berg, 1931 should be assigned to *Lethenteron* and we will provide a justification for its placement in *Eudontomyzon*.

The remaining three species recognized by Potter et al. (2015) as belonging to the genus *Lethenteron*, *Le. alaskense*, *Le. appendix*, and *Le. ninae*Naseka et al. 2009, will be included in a taxonomic key to the adults of the species of the genus that will also include the other species as established in this study. Artamonova et al. (2011) synonymized *Le. ninae* with *Le. camtschaticum*, but Tuniyev et al. (2016) confirmed the specific distinctiveness of the former. Although Tuniyev et al. (2016) showed that *Le. ninae* usually possesses a row of posterial teeth (complete or incomplete) characteristic of the genus *Lethenteron*, other morphological evidence (tricuspid middle endolateral, low number of trunk myomeres, straight longitudinal laminae, no velar wings) suggested that it should be assigned to *Lampetra*. However, they recommended the status quo until a total evidence cladistic analysis had been completed that incorporated parasitic and nonparasitic species and morphological and molecular characters to prevent re-assignment of a species to a different genus based on incomplete information.

## Materials and methods

Material examined follows the method of Renaud (2011) and for the gular pigmentation of Vladykov and Kott (1978a). TL refers to total length. All collection dates are according to the Gregorian calendar. We restricted our study to adults (i.e., metamorphosed individuals) because the diagnostic characters in the original descriptions and the type material of the three nominal subspecies and their synonyms were based on this life stage only.

### Abbreviations:

**CMNFI** Canadian Museum of Nature Fish Collection, Ottawa, Canada

**TGU**Tomsk State University, Tomsk, Russia;

**ZIN** (**ZISP** also used) Zoological Institute, Russian Academy of Sciences, St. Petersburg, Russia;

**ZMB**Museum für Naturkunde, Berlin, Germany.

### Type material (Fig. 1):

- ZMB 6475, 1 adult, 418.3 mm TL, neotype of Petromyzon marinus camtschaticus Tilesius, 1811 and lectotype of Petromyzon japonicus von Martens, 1868, Japan: Tokyo (originally Jeddo) and Yokohama, Honshu Island, Pacific Ocean basin, 1860–1863.
- ZMB 6476, 1 adult, 397.3 mm TL, paralectotype of Petromyzon japonicus, Japan: Tokyo (originally Jeddo) and Yokohama, Honshu Island, Pacific Ocean basin, 1860–1863.
- TGU 3 [no. 3699 in Ioganzen (1935b, table 3)], 1 adult, 182 mm TL, syntype of Petromyzon kessleri Anikin, 1905, Russia: Kirgizka River near Tomsk, Tom’ River system, Ob’ River drainage, Kara Sea basin, Arctic Ocean basin, 24 Dec. 1899, A. Neiland.
- TGU 9 [no. 3696 in Ioganzen (1935b, table 3)], 6 of 10 adults, 128–165 mm TL, syntypes of Petromyzon kessleri, Russia: near mouth of Tom’ River at Kozyulino, taken from a Common gull’s (Larus canus) digestive tract, Ob’ River drainage, Kara Sea basin, Arctic Ocean basin, 28 June 1903, G.E. Ioganzen.
- ZIN 12159, 8 of 10 adults, 281–374 mm TL, syntypes of Lampetra japonica septentrionalis Berg, 1931, Russia: Onega River at Podporozh’e, White Sea basin, Arctic Ocean basin, December 1901, N.A. Varpakhovskiy.
- ZMB 20638, 2 adults, 138.3–140.1 mm TL, syntypes of Lampetra mitsukurii Hatta, 1901, Japan: small watercourses on Hondo (= Honshu), Shikoku, Kyushu, and Hokkaido islands, Pacific Ocean basin.
- CMNFI 1984–274, 2 adults, 147.5–163.5 mm TL, paratypes of Lethenteron matsubarai Vladykov & Kott, 1978, Japan: Shokotsu River, Hokkaido Island, 44°22'N, 143°20'E, Sea of Okhotsk basin, Pacific Ocean basin, 1950–1952, T. Hikita.
- CMNFI 2008–59, 1 adult, 149.5 mm TL, paratype of Lethenteron ninae Naseka, Tuniyev & Renaud, 2009, Russia: Chakhtsutsyr Stream at Gumariya, Sochi District, Psou River drainage, Black Sea basin, 17–24 Dec. 2006, S.B. Tuniyev.

### Non-type material (Fig. 1):

#### Identified by Berg (1931) as *Lampetra japonica japonica*:

- ZIN 15188, 5 of 16 adults, 391.5–436.5 mm TL, Russia: Amur River, 6 km below Khabarovsk, Pacific Ocean basin, V.K. Soldatov.
- ZIN 23440, 1 adult, 170 mm TL, Russia: backwater of Kamchatka River near Ust-Kamchatsk, Pacific Ocean basin, 7 July 1908, P.J. Schmidt, beach seine.
- ZIN 23441, 1 adult, 187 mm TL, Russia: Kamchatka River near Ust-Kamchatsk, Pacific Ocean basin, June 1908, P.J. Schmidt, beach seine, lamprey descending towards the ocean.
- ZIN 23590, 2 adults, 154.5–198 mm TL, Russia: Kamchatka River near Ust-Kamchatsk, Pacific Ocean basin, 7 July 1909, P.J. Schmidt, lamprey descending towards the ocean.

#### Identified by Berg (1931) as *La. j. kessleri*:

- ZIN 6174, 3 adults, 202–212 mm TL, Russia: Ob’ River at Barnaul, Kara Sea basin, Arctic Ocean basin, Goebler.
- ZIN 6310, 1 adult, 187.5 mm TL, Russia: Ob’ River between Lake Teletskoye and Barnaul, Kara Sea basin, Arctic Ocean basin, 1876, Slovtsov.
- ZIN 6311, 1 adult, 144 mm TL, Russia: Irtysh River at Omsk, Ob’ River drainage, Kara Sea basin, Arctic Ocean basin, 1877, Poliakov.
- ZIN 7815, 1 adult, 186 mm TL, Kazakhstan: tributary to Irtysh River near Semipalatinsk (now Semey), Ob’ River drainage, Kara Sea basin, Arctic Ocean basin, 1887, Suvorcev.
- ZIN 14371, 1 adult re-identified as Le. camtschaticum because it possesses the silvery body coloration of an anadromous downstream migrant, 144 mm TL, Russia: Anadyr Estuary (Liman) at Novo-Mariinsk (now Anadyr), Bering Sea basin, Pacific Ocean basin, N. Gondatti.
- ZIN 14441, 1 adult, 122.5 mm TL, Russia: Yenisei River at Bazaikha, Kara Sea basin, Arctic Ocean basin, 29 June 1906.

#### Identified by Berg (1931) as *La. j. septentrionalis*:

- ZIN 7814, 1 adult, 351 mm TL, Russia: Tobol River at Tobolsk, Irtysh River system, Ob’ River drainage, Kara Sea basin, Arctic Ocean basin, 1887, I. Slovtsov.
- ZIN 8545, 1 adult, 301.5 mm TL, Russia: Vyg River at Soroka (now Belomorsk), White Sea basin, Arctic Ocean basin, 1886, Mizrakhanov.
- ZIN 20802, 1 adult, 334 mm TL, Russia: Shapkina River, Pechora River drainage, Barents Sea basin, Arctic Ocean basin, 5 Sept. 1921, G.D. Richter.

#### Supplementary topotype material of *La. j. septentrionalis* (Fig. 1):

- ZIN uncat., 11 adults, 290–347 mm TL, Russia: Onega River, 25 km upstream from its mouth, White Sea basin, Arctic Ocean basin, 1 Nov. 2011, A.P. Novoselov.

## Results

The morphometric, countable, shape, and pigmentary character states for the extant adult type material of *Petromyzon marinus camtschaticus*, *P. japonicus*, *P. kessleri*, and *La. j. septentrionalis* are given in Tables 1–3. The original descriptions of these nominal taxa are also an integral part of this section because they supplement our observations on the type material and because they were published in various languages (Latin, German, Russian, and English, respectively), they are presented below in English for ease of comparison. Additionally, as they span a period (1811–1931) when lamprey dentition nomenclature in particular was not yet standardized (see Vladykov and Follett 1967, Hubbs and Potter 1971), we used the current tooth nomenclature or added the equivalent name in parentheses.

The morphometric, countable, shape, and pigmentary character states in all non-type adults identified by Berg (1931) as *La. j. japonica*, *La. j. kessleri*, and *La. j. septentrionalis* available to us are given in Tables 4–6. Furthermore, the original descriptions of two species, *Petromyzon ernstii* and *P. dentex*, for which extant type material of neither could be found, are presented below in their English translation of the original German and Russian, respectively. Berg (1931) considered the former to be junior synonym of *La. j. japonica* and the latter, tentatively, as a junior synonym of *La. j. kessleri*. Berg (1931) considered *Ammocoetes aureus* to be a junior synonym of *La. j. japonica* and although its extant holotype was not examined, its original English description is presented below.

The morphometric, countable, shape, and pigmentary character states for extant adult type material of *La. mitsukurii* and *Le. matsubarai* are given in Tables 7–9 and their original English descriptions are also presented. Finally, the English translation of the original German descriptions of *La. m. minor* and *La. m. major* are presented.

### Original description of *Petromyzon marinus camtschaticus* Tilesius, 1811: 240–247, pl. IX, figs I, II.

The common name in Itelmen, formerly Kamchadal, a language spoken in Kamchatka, is Canaháisch. The written description and the drawings of the body (life size) and of the oral disc (enlarged) are based on a metamorphosed specimen collected 30 July 1804 (Julian calendar; 11 August 1804 Gregorian calendar) from marine waters in the harbor of Petropavlovsk-Kamchatsky, Russia. The drawing of the body in side view is thrown into three curves and by using a string along the curves we estimated the total length to be 308 mm. Disc length is ca. 16 mm (≈ 5.2% TL) and urogenital papilla length ca. 9.5 mm (≈ 3.1% TL). The two dorsal fins are separate; the interspace being ca. 22 mm. Caudal fin is lanceolate (= spade-like). Dentition: teeth are yellow; supraoral lamina with two bicuspid teeth separated by a wide bridge; two bicuspid endolaterals on each side; infraoral lamina with seven unicuspid teeth; transverse lingual lamina u-shaped, with seven blunt unicuspid teeth, the median one not enlarged in the figure, but noted as distinct in the text. The figure of the oral disc also shows a total of eleven anterials arranged in two rows; the first one consisting of five unicuspid teeth. Posterial and marginal teeth are not mentioned in the text, nor are they shown in the figure of the oral disc. Body pigmentation is not mottled, dorsal surface of head olive-brown, ventral body surface bluish-silvery, and tips of fins blackish; further on in the text he specifies that the posterior (= second) dorsal fin is blackish. The type specimen is lost, but Kottelat (1997) designated the lectotype of *Petromyzon japonicus* von Martens, 1868 as the neotype of *Petromyzon marinus camtschaticus* Tilesius, 1811, and it was studied (Tables 1–3).

**Table 1.** Morphometrics in adult types of *Petromyzon marinus camtschaticus* Tilesius, 1811, *Petromyzon japonicus* von Martens, 1868, *Petromyzon kessleri* Anikin, 1905, *Lampetra japonica septentrionalis* Berg, 1931 and adult topotypes of the latter. Numbers in parentheses are sample sizes.

	*Petromyzon marinus camtschaticus*ZMB 6475 (neotype)	*Petromyzon japonicus*ZMB 6476 (paralectotype)	*Petromyzon kessleri*TGU 3, TGU 9 (7 syntypes)^1^	*Lampetra japonica septentrionalis*ZIN 12159 (8 syntypes)	*La. j. septentrionalis*ZIN uncat. (11 topotypes)
Type locality	Tokyo and Yokohama, Japan	Tokyo and Yokohama, Japan	Tom’ and Kirgizka rivers, Russia	Onega River, Russia	Onega River, Russia
Total length (TL, mm)	418.3	397.3	128–182 (7)	281–374 (8)	290–347 (11)
Dorsal fin interspace (D_1_–D_2_, mm)	12.4	10.2	undetermined (7)	undetermined (8)	4.0–21.2 (11)
Intestinal diameter, mm	undetermined	undetermined	0.5 (3)	1.0–4.5 (8)	1.6–4.9 (11)
% **TL**					
Prebranchial length (d–B_1_)	9.8	9.3	9.1–13.7 (5)	9.0–10.9 (8)	9.6–10.6 (11)
Branchial length (B_1_–B_7_)	10.5	11.4	9.7–11.2 (4)	9.1–10.9 (8)	8.7–10.6 (11)
Trunk length (B_7_–a)	53.0	52.2	46.8–47.4 (2)	50.6–55.6 (8)	51.4–56.2 (11)
Cloacal slit length (a)	1.6	1.6	1.5 (1)	0.8–1.2 (8)	0.8–1.3 (11)
Tail length (a–C)	25.2	27.6	29.5 (1)	23.4–27.9 (8)	24.9–27.5 (11)
Disc length (d)	4.8	4.1	4.2–6.0 (5)	4.6–5.5 (8)	4.0–5.7 (11)
Prenostril length (d–n)	5.6	5.1	1.9–6.9 (5)	5.0–6.8 (8)	5.4–6.3 (11)
Snout length (d–O)	6.2	5.8	4.8–7.8 (5)	5.7–7.1 (8)	6.1–7.0 (11)
Eye length (O)	1.5	1.5	2.0–2.4 (5)	1.3–2.0 (8)	1.1–1.5 (11)
Postocular length (O–B_1_)	2.7	2.8	1.9–3.2 (5)	2.2–2.9 (8)	2.4–2.8 (11)
Interbranchial opening length (B_1_–B_2_)	1.4	1.2	1.3–1.6 (4)	1.1–1.5 (8)	1.2–1.5 (11)
Interocular width (I)	3.3	3.0	2.5–3.2 (5)	2.6–3.4 (8)	2.9–3.8 (11)
Urogenital papilla length	undetermined	0.7	0.5–0.8 (3)	0.0–0.8 (8)	0.4–0.7 (11)

^1^ Six of the seven syntypes (TGU 9) were collected from the digestive tract of a Common gull (*Larus canus*) and their condition was such that not all morphometrics could be measured.

**Figure 1.**
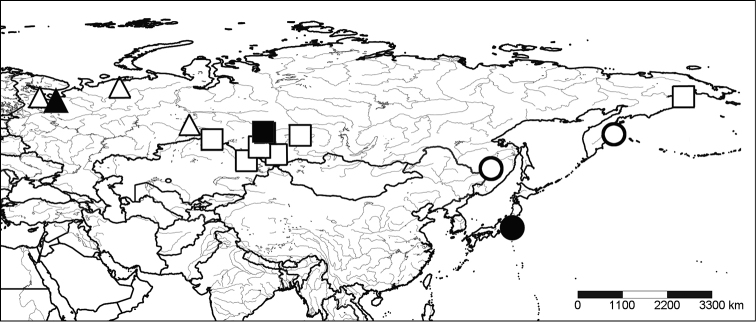
Geographic distribution of the lamprey genus *Lethenteron* in Eurasia based strictly on the examination of type material of *Petromyzon marinus camtschaticus* (●), *Petromyzon kessleri* (■), *Lampetra japonica septentrionalis* (▲) and ZIN material identified by Berg (1931) as *Lampetra japonica japonica* (○), *La. j. kessleri* (□), and *La. j. septentrionalis* (∆). Note that the easternmost record of *La. j. kessleri* from the Anadyr Estuary has been re-identified as *Le. camtschaticum*.

### Original description of *Petromyzon japonicus* von Martens, 1868: 3–5, pl. I, fig. 2.

**Figure 2.**
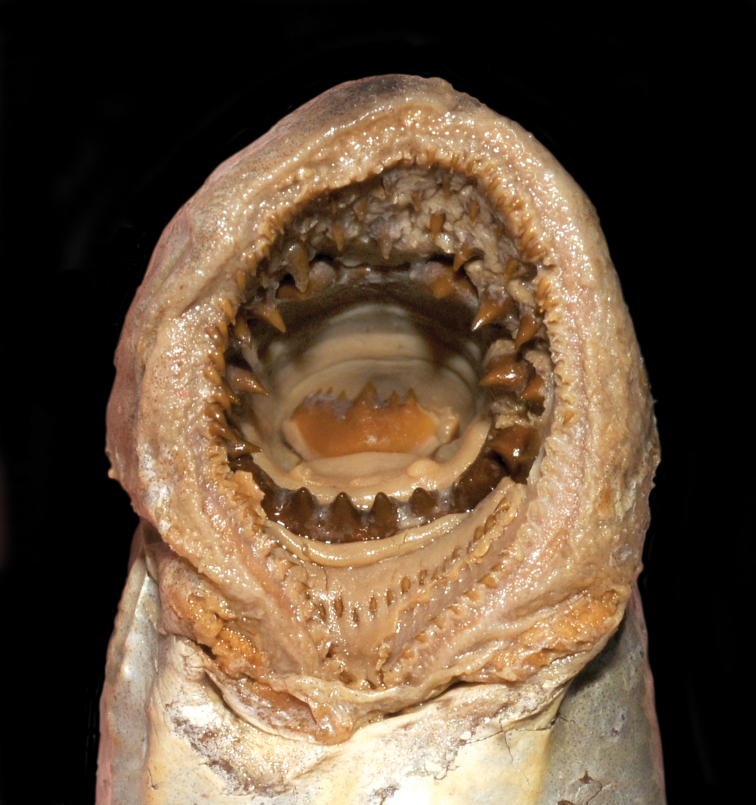
Oral disc of neotype of *Petromyzon marinus camtschaticus* and lectotype of *P. japonicus*, ZMB 6475, 418.3 mm TL.

The Japanese common name is Yats’-me-anango meaning eight-eyes-eel, in reference to the eye and seven branchial openings on the side of the body and eel-like body shape. The written description and the drawing of the oral disc (scale not provided) are based on an unspecified number of metamorphosed specimens collected between 1860 and 1863 from Tokyo (appeared as Jeddo) and Yokohama, Honshu Island, Japan. It is not clearly stated whether von Martens collected these himself or if they were obtained from the fish market. The habitat from which they came (i.e., freshwater, brackish or marine) is, therefore, not certain. Total length, 454 mm; snout length, 29 mm (= 6.4% TL). The two dorsal fins are separate, the interspace is 2.5 times the eye diameter (eye diameter not provided). Dentition (Fig. 2): row of similarly-sized, slender and pointed marginals; between the marginals and the infraoral lamina is a row of 16 small posterials (we counted 19 on the drawing); between the marginals and supraoral lamina are several larger teeth in a quincunx arrangement (the quincunx arrangement is not apparent on the drawing); supraoral lamina is crescent shaped, each side with a strong unicuspid tooth; three bicuspid endolaterals on each side (the drawing shows 1–2–2 on the left and 2–2–1–2 on the right side); infraoral lamina with six unicuspid teeth (we counted seven on the drawing), the two outermost larger (smaller on the drawing); transverse lingual lamina without a furrow, with a strong middle unicuspid tooth and four unicuspid teeth on each side (only three unicuspid teeth on each side were discernible on the drawing); longitudinal lingual laminae paired, narrow, with numerous cusps facing each other. Body pigmentation is slate gray on the dorsal surface and silvery on the lateral and ventral surfaces. One lectotype and one paralectotype was studied (Tables 1–3).

**Table 2.** Trunk myomeres, dentition, oral papillae and oral fimbriae in adult types of *Petromyzon marinus camtschaticus* Tilesius, 1811, *Petromyzon japonicus* von Martens, 1868, *Petromyzon kessleri* Anikin, 1905, *Lampetra japonica septentrionalis* Berg, 1931, and adult topotypes of the latter. Numbers in parentheses are frequencies of character states. Abbreviations: b, bicuspid; u, unicuspid.

	*Petromyzon marinus camtschaticus*ZMB 6475 (neotype)	*Petromyzon japonicus*ZMB 6476 (paralectotype)	*Petromyzon kessleri*TGU 3, TGU 9 (7 syntypes)^1^	*Lampetra japonica septentrionalis*ZIN 12159 (8 syntypes)	*La. j. septentrionalis*ZIN uncat. (11 topotypes)
Type locality	Tokyo and Yokohama, Japan	Tokyo and Yokohama, Japan	Tom’ and Kirgizka rivers, Russia	Onega River, Russia	Onega River, Russia
Trunk myomeres	75	70	70 (2), 73, 74, undetermined (3)	undetermined (8)	68, 71, 72 (4), 73, 74 (3), 76
Supraoral lamina	1u–1u	1u–1u	1u–1u (5), undetermined (2)	1u–1u (8)	1u–1u (11)
Endolateral formula	2–2–2 (2)	2–2–2 (2)	2–2–2 (9), 2–2–3, undetermined (4)	2–2–2 (14), 2–2–1 (2)	2–2–2 (22)
Infraoral lamina	1b4u1b	1b5u	1b4u1b (3), 1b5u1b (2), undetermined (2)	1b4u1b (8)	1b4u1b (10), 1b7u
Rows of anterials	2	2	2 (2), 3 (3), undetermined (2)	2 (6), 3 (2)	2 (4), 3 (7)
Rows of exolaterals	0 (2)	0 (2)	0 (10), undetermined (4)	0 (16)	0 (22)
Rows of posterials	1	1	1 (3), undetermined (4)	1 (8)	1 (11)
First anterial row	5u	3u	5u (5), undetermined (2)	3u (4), 4u (3), 5u	3u (9), 4u, 5u
First posterial row	20u	19u	24u (2), 29u, undetermined (4)	19u (3), 20u, 21u (2), 22u, 24u	18u, 19u, 20u (2), 21u, 22u (4), 23u (2)
Transverse lingual lamina	2u–I–6u	2u–I–3u	5u–I–7u, 7u–I–7u, undetermined (5)	4u–I–4u (2), 4u–I–5u, 6u–I–7u, 7u–I–6u, 7u–I–7u (2), 7u–I–8u	6u–I–5u, 6u–I–6u (2), 7u–I–6u (3), 7u–I–7u (2), 7u–I–8u (2), 8u–I–8u
Longitudinal lingual laminae	undetermined (2)	7u, 8u	undetermined (14)	undetermined (16)	9u (3), 10u (5), 11u, 12u (2), 13u, undetermined (10)
Oral papillae	undetermined	undetermined	undetermined (7)	13, 16 (3), 18 (4)	12, 14, 15 (2), 16, 18 (2), 21, undetermined (3)
Oral fimbriae	undetermined	undetermined	undetermined (7)	85, 91, 94, 97 (2), 98, 102, 103	88, 89 (2), 90, 92, 94 (2), 96, undetermined (3)

^1^ Six of the seven syntypes (TGU 9) were collected from the digestive tract of a Common gull (*Larus canus*) and their condition was such that not all counts could be made.

### Original description of *Petromyzon kessleri* Anikin, 1905: 10–15.

**Figure 3.**
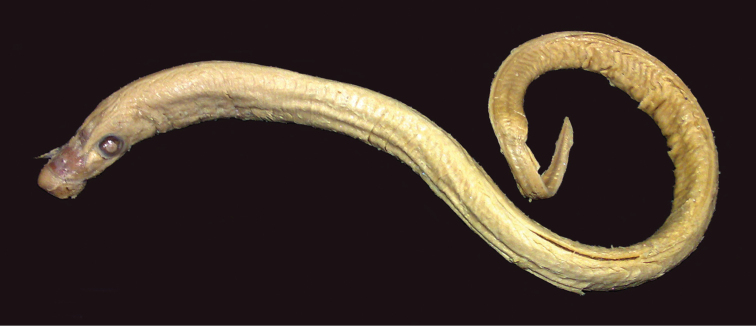
Syntype of *Petromyzon kessleri*, TGU 3 [no. 3699 in Ioganzen (1935b: table 3)], 182 mm TL.

The written description is based on 16 metamorphosed specimens (Fig. 3) collected in the Tom’ River and at the mouth of its tributary the Kirgizka River, Ob’ River drainage; both localities near Tomsk, Russia. Total length, 160–210 mm. Two dorsal fins; the interspace varying from 0 (touching at their bases with swelling at the site of contact) to 10 mm. The second dorsal fin is usually angular and 1.5–2 times higher than the rounded first dorsal fin. The second dorsal fin is continuous with the caudal fin. The caudal fin shape is rhomboid (= spade-like). Dentition: row of minute marginals; 20–25 anterials becoming progressively smaller towards the anterior end of the oral disc and arranged in staggered order in five rows; supraoral lamina with two large, either sharp, blunt or intermediate shape, usually dark yellow teeth separated by a bridge; three bicuspid endolaterals on each side; infraoral lamina with six or seven, usually six, exceptionally nine (Mean = 6.6), large, either sharp or blunt, usually dark yellow teeth, the lateralmost being bicuspid and the internal ones unicuspid; a single row of 20–25 small posterials; semi-circular transverse lingual lamina with a large, sharp median tooth and eight or nine smaller teeth on each side; pair of longitudinal lingual laminae with an unspecified number of fine teeth facing each other. Body pigmentation is pale brown, ash gray or black on the dorsal surface, white or yellowish on the ventral surface and the end of the tail is dark brown. The line of demarcation between the dorsal and ventral pigmentation is not always distinct. The dorsal and caudal fins are white or yellowish except that the apex of the second dorsal fin is ash gray and the tip of the caudal fin is dark brown. Seven syntypes were studied (Tables 1–3).

**Table 3.** Shape and pigmentary characters in adult types of *Petromyzon marinus camtschaticus* Tilesius, 1811, *Petromyzon japonicus* von Martens, 1868, *Petromyzon kessleri* Anikin, 1905 and *Lampetra japonica septentrionalis* Berg, 1931 and adult topotypes of the latter. Numbers in parentheses are frequencies of character states. Pigmentation coverage as follows: -, absent to < 1%; +, 1 to < 25%; +++, ≥ 75%.

	*Petromyzon marinus camtschaticus*ZMB 6475 (neotype)	*Petromyzon japonicus*ZMB 6476 (paralectotype)	*Petromyzon kessleri*TGU 3, TGU 9 (7 syntypes)^1^	*Lampetra japonica septentrionalis*ZIN 12159 (8 syntypes)	*La. j. septentrionalis*ZIN uncat. (11 topotypes)
Type locality	Tokyo and Yokohama, Japan	Tokyo and Yokohama, Japan	Tom’ and Kirgizka rivers, Russia	Onega River, Russia	Onega River, Russia
Caudal fin shape	spade-like	spade-like	undetermined (7)	spade-like (8)	spade-like (11)
**Pigmentation**					
Caudal fin	+++	+++	undetermined (7)	+++ (7), undetermined	+++ (10), undetermined
Second dorsal fin	with blotch	with blotch	undetermined (7)	no blotch, with blotch (6), undetermined	with blotch (10), undetermined
Lateral line neuromasts	undetermined	undetermined	undetermined (7)	pigmented, undetermined (7)	unpigmented (2), pigmented, undetermined (8)
Gular	undetermined	undetermined	undetermined (7)	- (7), undetermined	- (7), + (2), +++, undetermined

^1^ Six of the seven syntypes (TGU 9) were collected from the digestive tract of a Common gull (*Larus canus*) and their condition was such that no characters could be evaluated.

### Original description of *Lampetra japonica septentrionalis* Berg, 1931: 93, 100–102, pl. V, fig. 4.

**Figure 4.**
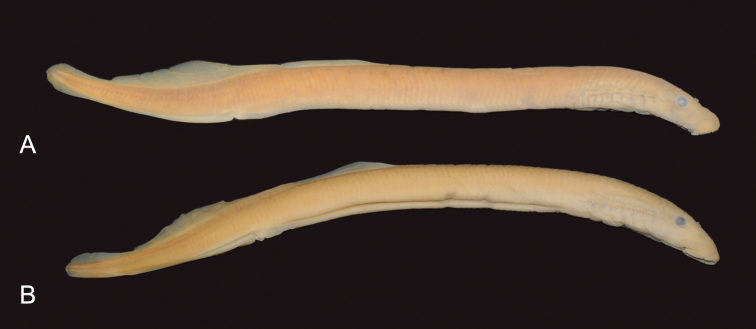
Syntypes of *Lampetra mitsukurii*, ZMB 20638, 138.3 (a) and 140.1 (b) mm TL.

The written description is based on ten metamorphosed specimens from the Onega River at Podporozh’e, White Sea basin, Russia, collected December 1901 by N.A. Varpakhovskiy (ZIN 12159). The common name given is Arctic lamprey. Total length, 284–377 mm (Mean = 327 mm). Dentition: infraoral lamina with six sharp teeth (Berg’s notation is 1+4+1, but he does not explain the reason for the distinction between the types of teeth). Berg (1931) provides a drawing of the oral disc of a non-type specimen collected from the type locality in December 1929 (Berg 1931: pl. V, fig. 4) and it becomes clear that 1+4+1 means four unicuspid teeth flanked on each side by one enlarged bicuspid tooth. This is the condition (1b4u1b) of all eight syntypes examined in this study (Table 2). The drawing also shows a row of marginals, two rows of anterials, the first row with three unicuspid teeth, one row of posterials with 19 unicuspid teeth, no exolaterals on either side, supraoral lamina with two unicuspid teeth separated by a wide bridge, three bicuspid endolaterals on each side, transverse lingual lamina with an enlarged median cusp flanked on the left side by six unicuspid teeth and on the right by at least five. Additional characteristics reported by Berg (1931) in non-type specimens from the Onega River are an infraoral lamina count of 1+6+1 and four adult individuals with their ventral body surface mottled brown and one with the body entirely black. In a specimen from the Tura River the infraoral lamina count is 1+5+1 and in a specimen from the Vyg River a third cusp is placed asymmetrically between the two larger cusps on the supraoral lamina. Based on material examined and literature, Berg (1931) determined that the distribution of *La. j. septentrionalis* occurs from the White Sea basin to the Ob’ River drainage. The specific rivers listed from west to east are: Umba (only apparently because based on an ammocoete), Vyg, Onega, Severnaya Dvina, Pesha, Shapkina (Pechora River drainage), Tura, Tobol, Irtysh, Tom’, and also the Gulf of Ob’. The shortest adult is 227 mm TL from the Vyg River at Soroka (= Belomorsk) and the longest is 430 mm TL from the Tura River at Tyumen. In the Onega River it is fished commercially. Berg (1931) diagnosed this subspecies from *La. j. japonica* based on the smaller length of its upstream migrants (227–430 mm TL versus 352–625 mm TL, respectively) and its lower fecundity: 24,086–25,144 eggs in two females, 335–339 mm TL, collected from the Onega River at the end of November 1929 compared to 80,825–107,015 eggs in six females, 403–492 mm TL, respectively (the TL of the female with the lowest fecundity was unknown), collected from Tneyvakh, below Nikolayevsk-on-Amur, lower Amur River, Russia between September and 20 November 1929. Berg (1931) diagnosed this subspecies from *La. j. kessleri* based on the latter having few eggs (no specific numbers given) and adults only reaching a maximum of 260 mm TL. Eight syntypes were studied (Tables 1–3).

**Table 4.** Morphometrics in adults identified by Berg (1931) as *Lampetra japonica japonica* (von Martens, 1868), *La. j. kessleri* (Anikin, 1905), and *La. j. septentrionalis* Berg, 1931. Numbers in parentheses are sample sizes.

	*Lampetra japonica japonica*		*Lampetra japonica kessleri*				*Lampetra japonica septentrionalis*		
Locality	Amur River	Kamchatka River	Ob’ River	Irtysh River system	Yenisei River	Anadyr Estuary	Tobol River	Vyg River	Shapkina River
ZIN catalogue no.	15188	23440, 23441, 23590	6174, 6310	6311, 7815	14441	14371^1^	7814	8545	20802
Total length (TL, mm)	391.5–436.5 (5)	154.5–198 (4)	187.5–212 (4)	144–186 (2)	122.5 (1)	144 (1)	351 (1)	301.5 (1)	334 (1)
Intestinal diameter, mm	1.0–2.5 (5)	2.5–4.5 (4)	0.7–1.3 (3), undetermined	0.7, undetermined	0.5	0.5	5.0	2.5	3.0
% **TL**									
Prebranchial length (d–B_1_)	9.4–10.8 (5)	12.6–14.2 (4)	9.6–12.0 (4)	10.8–11.8 (2)	11.4	12.8	11.0	10.6	9.9
Branchial length (B_1_–B_7_)	10.0–10.9 (5)	9.4–10.4 (4)	8.2–9.6 (4)	9.0–9.1 (2)	10.6	9.4	9.3	10.9	9.6
Trunk length (B_7_–a)	54.1–55.3 (5)	46.9–51.8 (4)	49.1–51.2 (4)	47.3–47.9 (2)	51.4	50.7	49.7	50.9	50.6
Cloacal slit length (a)	0.7–0.9 (5)	0.8–1.2 (4)	0.5–1.2 (4)	0.7–1.1 (2)	0.8	0.7	0.6	1.0	0.7
Tail length (a–C)	24.1–25.9 (5)	24.2–27.8 (4)	29.2–30.3 (4)	30.2–31.7 (2)	26.5	27.4	28.1	25.5	29.0
Disc length (d)	4.6–5.4 (5)	6.4–7.1 (4)	4.0–6.7 (4)	5.6–5.9 (2)	4.1	4.5	5.8	5.6	4.6
Prenostril length (d–n)	5.6–6.8 (5)	7.2–8.1 (4)	5.2–7.2 (4)	6.2–6.4 (2)	6.1	6.6	6.0	5.6	5.5
Snout length (d–O)	5.6–7.0 (5)	8.0–9.1 (4)	5.4–7.7 (4)	6.7–6.9 (2)	6.1	7.6	7.1	6.6	6.4
Eye length (O)	1.2–1.4 (5)	2.1–2.5 (4)	1.7–2.4 (4)	2.7–2.8 (2)	2.4	2.4	1.6	1.8	1.2
Postocular length (O–B_1_)	2.6–2.9 (5)	2.7–3.0 (4)	2.0–2.7 (4)	2.4 (2)	3.3	3.1	2.3	2.3	2.7
Interbranchial opening length (B_1_–B_2_)	1.1–1.4 (5)	1.2–1.3 (4)	1.1–1.6 (4)	1.0–1.1 (2)	1.6	1.4	1.1	1.3	1.2
Interocular width (I)	3.2–3.8 (5)	3.2–3.7 (4)	2.2–3.7 (4)	2.7–2.8 (2)	3.7	2.1	2.7	3.6	3.1
Urogenital papilla length	0.0–0.4 (5)	0.5–0.8 (4)	0.0–1.0 (4)	0.7–1.1 (2)	1.2	0.0^2^	0.6	0.7	0.6

^1^ Re-identified as *Lethenteron camtschaticum* because it possesses the silvery body coloration of an anadromous downstream migrant. ^2^ The urogenital papilla did not protrude beyond the cloacal slit.

### Original description of *Petromyzon ernstii* Dybowski, 1872: 220.

The written description is based on a 310 mm TL metamorphosed specimen from the mouth of the Amur River, Russia. At the periphery of the suctorial disc is a series of small pointed teeth (= marginals); a second more centrally-located circular series of teeth consists in its lower zone of a row of small pointed teeth (= posterials), in its central zone, on each side, of three transversely-positioned tooth plates (= endolaterals), of which only the top two are bicuspid (the condition of the bottom endolateral is not given), and in its upper zone of two rows of more pointed conical teeth (= anterials) numbering eleven. Maxillary arch (= supraoral lamina) with two strong, sharp canine teeth. Mandibular arch (= infraoral lamina) with an average (therefore, the description involved more than one individual, but only one measuring 310 mm TL is mentioned) of four smaller-pointed teeth, and on either side a strong bicuspid tooth. Tongue crescent bar (= transverse lingual lamina) with 19 teeth; a narrow, weakly convex-concave (= parenthesis-shaped) bar (longitudinal lingual lamina) with 12 pointed teeth at both extremities of the crescent bar. First dorsal fin separated from the higher second dorsal fin by a wide gap. Body ash gray colored above and silvery below. The whereabouts of the type specimen is unknown and is presumed lost.

**Table 5.** Trunk myomeres, dentition, oral papillae and oral fimbriae in adults identified by Berg (1931) as *Lampetra japonica japonica* (von Martens, 1868), *La. j. kessleri* (Anikin, 1905), and *La. j. septentrionalis* Berg, 1931. Numbers in parentheses are frequencies of character states. Abbreviations: b, bicuspid; u, unicuspid.

	*Lampetra japonica japonica*		*Lampetra japonica kessleri*				*Lampetra japonica septentrionalis*		
Locality	Amur River	Kamchatka River	Ob’ River	Irtysh River system	Yenisei River	Anadyr Estuary	Tobol River	Vyg River	Shapkina River
ZIN catalogue no.	15188	23440, 23441, 23590	6174, 6310	6311, 7815	14441	14371^1^	7814	8545	20802
Trunk myomeres	undetermined (5)	73, undetermined (3)	76–77 (2), undetermined (2)	75, undetermined	76	undetermined	undetermined	undetermined	undetermined
Supraoral lamina	1u–1u (5)	1u–1u (4)	1u–1u (3),	1u–1u (2)	1u–1u	1u–1u	1u–1u	1u–1u	1u–1u
			1u–1u–1u						
Endolateral formula	2–2–2 (10)	2–2–2 (8)	2–2–2 (8)	2–2–2 (3), 2–2–?	2–2–2 (2)	2–2–2 (2)	2–2–2 (2)	2–2–2 (2)	2–2–2 (2)
Infraoral lamina	1b4u1b (3),	1b4u1b, 1b5u1b	1b4u1b, 1b5u1b	1b4u1b, 1b5u1b	1b3u1b	1b5u1b	1b4u1b	1b4u1b	1b4u1b
	1b5u1b,	(2),	(3)						
	1b6u1b	1b1u1b2u1b							
Rows of anterials	2, 3 (4)	2 (3), 3	3 (4)	2 (2)	2	2	3	2	2
Rows of exolaterals	0 (10)	0 (8)^2^	0 (8)^3^	0 (3), undetermined	0 (2)	0 (2)	0 (2)^4^	0 (2)	0 (2)
Rows of posterials	1 (5)	1(3), 2^5^	1 (4)	1 (2)	1	1	1	1	1
First anterial row	3u (3), 4u (2)	3u (3), 5u	3u (2), 4u, 6u	4u, undetermined	2u	5u	3u	4u	1u
First posterial row	18u, 19u (2), 20u, 22u	20u, 21u, 22u, 7u2b13u	25u, 28u, 31u, undetermined	21u, ≈30u	18u	undetermined	23u	20u	21u
Transverse lingual lamina	6u–I–7u,	8u–I–7u,	7u–I–7u (2),	6u–I–5u,	undetermined	8u–I–8u	8u–I–9u	?–I–?	4u–I–4u
	7u–I–6u,	?–I–8u,	7u–I–8u,	6u–I–6u					
	7u–I–7u (2),	undetermined (2)	9u–I–7u						
	?–I–?								
Longitudinal lingual laminae	undetermined (10)	undetermined (8)	12u (2), undetermined (6)	7u (2), ≈13u (2)	undetermined	undetermined	undetermined	undetermined	undetermined
Oral papillae	16, 17 (2), 22, undetermined	18, 20, undetermined (2)	17, 26, undetermined (2)	undetermined (2)	undetermined	undetermined	undetermined	undetermined	19
Oral fimbriae	96, 100, 111, 126, undetermined	92, undetermined (3)	≈110, 121, 122, undetermined	>120, undetermined	≈82	undetermined	undetermined	undetermined	98

^1^ Re-identified as *Lethenteron camtschaticum* because it possesses the silvery body coloration of an anadromous downstream migrant. ^2^ One exolateral tooth on the right side and one on the left side in the middle of the lateral field between the first and second endolaterals in one specimen. ^3^ One exolateral tooth on the right side next to the marginals between the first and second endolaterals in one specimen. ^4^ One exolateral tooth on the left side between the third endolateral and the marginals. ^5^ Two unicuspid teeth in the second row of posterials.

**Table 6.** Shape and pigmentary characters in adults identified by Berg (1931) as *Lampetra japonica japonica* (von Martens, 1868), *La. j. kessleri* (Anikin, 1905), and *La. j. septentrionalis* Berg, 1931. Numbers in parentheses are frequencies of character states. Pigmentation coverage as follows: -, absent to < 1%; +, 1% to < 25%; ++, 25% to < 75%; +++, ≥ 75%.

	*Lampetra japonica japonica*		*Lampetra japonica kessleri*				*Lampetra japonica septentrionalis*		
Locality	Amur River	Kamchatka River	Ob’ River	Irtysh River system	Yenisei River	Anadyr Estuary	Tobol River	Vyg River	Shapkina River
ZIN catalogue no.	15188	23440, 23441, 23590	6174, 6310	6311, 7815	14441	14371^1^	7814	8545	20802
Caudal fin shape	spade-like (2), undetermined (3)	spade-like (4)	spade-like (3), undetermined	spade-like (2)	spade-like	undetermined	spade-like	spade-like	spade-like
**Pigmentation**:									
Caudal fin	+++ (4), undetermined	+++ (4)	+++, undetermined (3)	+++ (2)	+++	undetermined	undetermined	+++	+++
Second dorsal fin	with blotch (5)	with blotch (4)	with blotch, undetermined (3)	with blotch, undetermined	with blotch	with blotch	undetermined	with blotch	with blotch
Lateral line neuromasts	undetermined (5)	unpigmented (2), undetermined (2)	unpigmented, undetermined (3)	unpigmented, undetermined	undetermined	undetermined	undetermined	undetermined	unpigmented
Gular	- (4), +	-, + (2), ++	- (2), +, undetermined	+, +++	+++	undetermined	-	+	-

^1^ Re-identified as *Lethenteron camtschaticum* because it possesses the silvery body coloration of an anadromous downstream migrant.

### Original description of *Petromyzon dentex* Anikin, 1905: 15–17.

The written description is based on two metamorphosed specimens ca. 160 mm long collected in the summer of 1903 at the mouth of the Yenisei River near Gol’chikha, Russia. These specimens were presumed to have come from the digestive system of a bird or a fish because their mucous layer was absent and all the fins were destroyed, leaving only occasional shreds. The dorsal body surface was black and the ventral surface yellowish white, with a sharp boundary between the two areas. The posterior end of the body and the tip of the caudal fin were black. Dentition is identical to that of *P. kessleri* in terms of number and arrangement of teeth in the upper jaw (= supraoral lamina), lower jaw (= infraoral lamina), paired lateral teeth (= endolaterals) and all the fine teeth (= anterials and posterials), but resembles that of *Petromyzon* (= *Lampetra*) *fluviatilis* in the teeth being larger and sharp. It may be assumed that the Siberian lampreys are represented by two forms, parallel to the European *Petromyzon* (= *Lampetra*) *planeri* and *P. fluviatilis*; *P. planeri* corresponds to *P. kessleri* and *P. fluviatilis* corresponds to *P. dentex*. The teeth are brown. The number of teeth on the infraoral lamina is not the same in the two specimens; one has six teeth, and the other has eight teeth, with two of the inside teeth being incompletely separated. This indicates that each of these incompletely divided teeth originate from a single tooth, resulting in the total number of eight instead of six teeth. The outermost teeth of the infraoral lamina have two inconspicuous cusps (i.e., bicuspid) in both specimens. There are 17–20 anterials arranged in four oblique rows and these teeth decrease in size from the center to the periphery of the oral disc. The row of small posterial teeth extends from one side to the other, its ends reaching the lower lateral pairs of teeth (= endolaterals). The two syntypes are presumed lost because Ioganzen (1935b) could not find them at TGU where they had been deposited and nor could AMN during his visit in 2011.

### Original description of *Ammocoetes aureus* Bean, 1881: 159.

The written description is based on one metamorphosed specimen (holotype, USNM 21524) 15 inches (= 381 mm) TL collected in 1877 (Bean 1882) from the Yukon River at Anvik (63°N, 160°W), Alaska, U.S.A. Dentition: maxillary (= supraoral lamina) with two cusps and mandibular (= infraoral lamina) with seven, the lateralmost one on each side enlarged. Eye length (O), nearly twice the width of the largest branchial opening, but the latter not given. Head or prebranchial length (d-B_1_), 9.7% of TL; tail length (a-C), 25% of TL (uncertain proportion because not clear if the measurement was taken from the anterior or posterior edge of the cloaca). Two dorsal fins with an interspace of ca. 24 mm. Coloration (in alcohol) of the back plumbeous, sides and belly golden yellow (hence, the specific name), and underside of the head and branchial region silvery. The collector, L.M. Turner, noted that the species was extremely abundant and used for food. The holotype was not studied.

### Original description of *Lampetra mitsukurii* Hatta, 1901: 22–24.

The written description is based on an unspecified number of metamorphosed specimens measuring 80–156 mm TL and collected from small watercourses (streamlets between fields, springs, and small canals) on Hondo [= Honshu], Shikoku, Kyushu, and Hokkaido islands, Japan. Hatta (1901) states that the new species is distinct from *La. japonica* measuring 390–507 mm TL that occurs in waterbodies (rivers, lakes, and ponds) of Honshu and Hokkaido islands, Sea of Japan basin, Japan. Additionally, it is diagnosable from *La. japonica* by having a more protruded suctorial disc; less prominent and more obtuse teeth; an unspecified lesser number of teeth in the series outside the mandibular tooth-plate (row of posterial teeth); the cusps at the lateral extremities of the mandibular tooth-plate (infraoral lamina) not bifurcated; the first and second dorsal fins are not separated by an interspace, only a notch; the anal fin (= fin-like fold) is of considerable height in females during the spawning season; the labial tentacles (= oral fimbriae) are mostly palmate; the skin is dark brown with faint irregular spots. Hatta (1901) notes that the two most important diagnostic characters are the smaller body size (80–156 versus 390–507 mm TL) and the unicuspid (versus bicuspid in *La. japonica*) lateralmost teeth on either side of the infraoral lamina. He suggests that the American brook lamprey, *La. wilderi* is probably the closest relative to *La. mitsukurii*. Two syntypes were studied (Fig. 4, Tables 7–9).

### Original descriptions of *Lampetra mitsukurii minor* Hatta, 1911: 263–266, 268, pl. IX, figs 3, 4, 7, 8 and *La. m. major* Hatta, 1911: 266–268, pl. IX, figs 1, 2, 5, 6.

The written description of *Lampetra mitsukurii minor* is based on an unspecified number of metamorphosed specimens measuring 80–165 mm TL from 14 localities across Japan as follows: Sapporo (Hokkaido Island), Tsuyama, Takayama, Akita, Aganogawa, Tamagawa, Kawagoye, Yamagata, Yamaguchi, Sakura, Hamamatsu, Gifu (eleven localities on Honshu Island), Matsuyama (Shikoku Island), Kumamoto (Kyushu Island). It is supplemented by drawings of the body of a spawning male and a spawning female from Sapporo in side-view, as well as close-ups of their cloacal regions (Hatta 1911: figs 3, 4 and 7, 8, respectively). The written description of *La. m. major* is based on an unspecified number of metamorphosed specimens measuring 350–410 mm TL from Sapporo (Hokkaido Island); the maximum TL in the range having been determined from Hatta (1911: fig. 1). It is supplemented by drawings of the body of a spawning male and a spawning female in side-view, as well as close-ups of their cloacal regions (Hatta 1911: figs 1, 2 and 5, 6, respectively). The males of both subspecies possess well-developed urogenital papillae and the females of both subspecies possess well-developed anal fin-like folds. Both subspecies are diagnosable from Japanese *La. japonica* because the latter is larger (450–507 mm TL), not externally sexually dimorphic, its supraoral and infraoral laminae cusps are sharp instead of blunt and its intestine relatively thick instead of thread-like. Additionally, while *La. m. major* arrives on its spawning grounds at the end of April, *La. japonica* arrives on its spawning grounds in late May or early June. *La. m. major* is sympatric with *La. m. minor* at Sapporo and is often found attached to the latter, but is allopatric with *La. japonica*. No type material was studied.

**Table 7.** Morphometrics in adult types of *Lampetra mitsukurii* Hatta, 1901 and *Lethenteron matsubarai* Vladykov & Kott, 1978. Numbers in parentheses are sample sizes.

	*Lampetra mitsukurii*	*Lethenteron matsubarai*
	ZMB 20638 (2 syntypes)	CMNFI 1984-274 (2 paratypes)
Type locality	Japan	Shokotsu River, Hokkaido Island, Japan
Total length (TL, mm)	138.3–140.1	147.5–163.5
Dorsal fin interspace (D_1_–D_2_, mm)	0.0 (2)	0.0 (2)
Intestinal diameter, mm	undetermined (2)	0.5, undetermined
% **TL**		
Prebranchial length (d–B_1_)	9.9–10.6	11.9(2)
Branchial length (B_1_–B_7_)	10.7–10.9	8.9–9.2
Trunk length (B_7_–a)	49.1–51.0	48.1–48.6
Cloacal slit length (a)	0.8–1.1	0.7–1.2
Tail length (a–C)	27.6–28.8	30.2–31.2
Disc length (d)	4.9–5.2	5.4–5.5
Prenostril length (d–n)	4.8–5.3	5.8–6.1
Snout length (d–O)	5.9–6.7	7.0–7.1
Eye length (O)	1.6–1.7	1.5–2.4
Postocular length (O–B_1_)	2.6–2.7	3.1 (2)
Interbranchial opening length (B_1_–B_2_)	1.2–1.3	1.2–1.4
Interocular width (I)	3.3–3.6	2.7–3.1
Urogenital papilla length	undetermined (2)	0.0–0.6

### Original description of *Lethenteron matsubarai* Vladykov & Kott, 1978: 1792–1800.

The written description is based on seven metamorphosed specimens (holotype + six paratypes) measuring 150–174 mm TL collected in 1950–1952 from Shokotsu River, Hokkaido Island, Japan (44°22'N, 143°20'E). It is supplemented by photographs of the holotype (whole body in side-view and oral disc) and paratypes (intestine and velar apparatus). *Lethenteron matsubarai* is diagnosable from five metamorphosed *Le. japonicum* (= *Le. camtschaticum*) collected sympatrically and measuring 172–372 mm TL by being nonparasitic (intestinal diameter < 1 mm versus 3.5 mm), having weaker dentition (poorly cornified, pale yellow blunt cusps versus strongly cornified, orange sharp cusps) and by the absence of pigmentation versus dark pigmentation on the second dorsal and caudal fins. Additionally, disc length and eye diameter, as a percentage of TL, was respectively, 4.4–5.3 and 1.7–2.3 in *Le. matsubarai* measuring 150–174 mm TL versus 5.3–6.0 and 2.6–3.2 in sympatric *Le. camtschaticum* measuring 172–193 mm TL. Dentition: supraoral lamina with one cusp at each end; three bicuspid endolaterals on each side; infraoral lamina with six cusps; single row of 18–23 unicuspid posterials; transverse lingual lamina with an enlarged median cusp and up of eleven cusps in total. Body pigmentation in 4–5% formalin is light brown on back and sides and very light brown on ventral aspect. Trunk myomeres are 66–70 and velar tentacles seven or eight. Vladykov and Kott (1979b) provided an amended caption to a figure in the original description (Vladykov and Kott 1978b: fig. 4). Two paratypes were studied (Tables 7–9).

## Discussion

### One or multiple species

Hubbs (1925) proposed that what he called *Entosphenus mitsukurii* was a degenerate, dwarf, brook lamprey derived from anadromous, parasitic *Entosphenus japonicus* (= *Le. camtschaticum*). Whether morphologically-similar (other than maximum adult size attained), but trophically distinct paired subspecies or species sensu Zanandrea (1959) or stem-satellite species sensu Vladykov and Kott (1979a) [reviewed and updated by Salewski (2003)] represent distinct taxa, or a single trophically-plastic taxon sensu McPhail and Lindsey (1970), Yamazaki et al. (1998), Sawatzky et al. (2007), Kucheryavyi et al. (2007a, 2007b), Kucheryavyy et al. (2010a, 2010b, 2016), April et al. (2011), Nazarov et al. (2011), Yamazaki et al. (2011), Makhrov et al. (2013), Yamazaki and Nagai (2013), Kucheryavyy (2014), Artamonova et al. (2015), Makhrov and Popov (2015), and Yamazaki and Goto (2016) needs to be treated on a case by case basis as there appears to be a continuum of differentiation within lampreys [see reviews by Docker (2009) and Docker and Potter (2019)]. Between 17–21 June 1972 Savvaitova and Maksimov (1978) observed communal spawning of two forms (large and small) of *La. japonica* (= *Le. camtschaticum*) in Levyy Kolkalvayam River, tributary to Utkholok River, Sea of Okhotsk basin, western Kamchatka, Russia. The communal redds of large spawners (230–320 mm total length) and small spawners (100–140 mm total length) were located 60–80 km from the sea while redds consisting of small spawners only were located both closer to the coast and further upriver. Savvaitova and Maksimov (1978) proposed that the large spawners are the anadromous form and the small spawners the precociously mature resident freshwater form of *Le. camtschaticum*. Gritsenko (2002) also reported communal spawning of *Le. japonicum* (= *Le. camtschaticum*) (340–570 mm TL) and what he identified as *Le. kessleri* (< 230 mm TL), but could belong to another species (see below ‘Sympatric parasitic and nonparasitic taxa’, page 40) in the Tym’ River, Sakhalin Island, Sea of Okhotsk basin, Russia. However, he concluded that the two species were distinct because, in addition to their difference in total lengths, their trunk myomeres were non-overlapping by over 90%. In their review of lampreys in the Eurasian Arctic, encompassing over 70 rivers from the basins of the Barents, White, Kara, Laptev, East Siberian, Chukchi, and Bering seas, Makhrov et al. (2013) determined that *Le. camtschaticum* comprised both anadromous parasitic and resident nonparasitic forms and considered the latter form, represented by *Le. kessleri*, to be a junior synonym. Kucheryavyi et al. (2007a) had previously treated sympatric lampreys in the Utkholok River drainage possessing three distinct modes of life [i.e., normally-maturing anadromous lampreys feeding as adults, early-maturing (i.e., praecox form) anadromous lampreys that spend up to a year at sea, presumably for feeding, although this is not explicitly stated, but in Savvaitova et al. (2007) and Kucheryavyy et al. (2010b) it is, and freshwater-resident lampreys non-feeding as adults] as belonging to a single species, *Le. camtschaticum*. Kucheryavyi et al. (2007a, 2010b) proposed a mechanism to explain the co-existence of these morphologically-similar, other than mature adult total length attained (i.e., respectively, 174–350 mm, 145–220 mm, and 100–165 mm), and synchronously spawning lampreys. They suggested that what determined the adult mode of life was the larval diet, with the larvae that switch from their usual diet of organic detritus and algae to an energy-rich diet of decomposing carcasses of Pacific salmons (*Oncorhynchus* spp.) forego feeding as an adult and become residents, while those that fed only on organic detritus and algae begin feeding post-metamorphosis in fresh water and later at sea before returning to fresh water for spawning. Unfortunately, this does not explain why there are normally maturing and praecox anadromous forms (Renaud 2011). Additionally, while decaying salmonids may be available in this particular river drainage, this is not the case in other parts of Russia and in Kazakhstan where nonparasitic lamprey occur thousands of kilometers from the sea, namely the upper Ob’ and Irtysh rivers, respectively (Romanov et al. 2017). Kucheryavyy et al. (2010a) also suggested that size differences in spawning individuals of *Le. camtschaticum* was not a barrier to successful spawning in that species. In the Kolkavayam River, Utkholok River drainage, communal and paired matings of different size individuals of all three forms (i.e., means of 270–280 mm, 170 mm, ≈130 mm TL for anadromous, praecox-anadromous, and resident, respectively) in various combinations during spawning ensure what Kucheryavyy et al. (2010a) refer to as evolutionary stasis. Kucheryavyi et al. (2007b) had previously reported on six so-called spawning balls of *Le. camtschaticum* in the Utkholok River drainage that varied in size from six to 44 individuals, three of which (six, seven, 43 individuals) consisted of the resident form only and the other three (eight, 27, 44 individuals) consisted of a mixture of anadromous and resident forms in which the resident form was always predominant, constituting respectively, 62, 85, and 98% of individuals. On the other hand, Mateus et al. (2013) found 166 fixed allelic differences, 12 of which were assigned to genes, some of them controlling migratory behavior (i.e., anadromy vs. freshwater residency), between the species pair *La. fluviatilis* (anadromous parasitic) and *La. planeri* (resident nonparasitic) collected sympatrically at a spawning site in Portugal; thus indicating reproductive isolation. While such a study has not been conducted on *Le. camtschaticum* and its nonparasitic derivatives, we treat lampreys with different trophic modes of life as distinct species until this hypothesis has been falsified. Renaud et al. (2009) proposed common garden experiments as a way to elucidate this conundrum. Interestingly, preliminary studies by Yamazaki et al. (2011) and Yamazaki and Nagai (2013) indicate significant differences in allelic frequencies of polymorphic nuclear microsatellite loci between anadromous and what they characterize as fluvial nonparasitic landlocked (10 dams separating them from the sea) populations of *Le. camtschaticum* in Japan, and this may represent the first step in lamprey speciation. However, the results of these studies must be taken with caution because the two nonparasitic populations of *Le. camtschaticum* identified were represented only by ammocoetes (Ina and Tateiwa rivers, Agano River system, Honshu Island). Yamazaki and Goto (2000a) had previously proposed that in Japan speciation of lampreys of the genus *Lethenteron* had occurred from ancestral populations of anadromous parasitic *Le. japonicum* (= *Le. camtschaticum*) via precocious dwarf individuals leading independently to distinct nonparasitic *Le. kessleri* and what they called a northern form of *Le. reissneri* (see below ‘Taxonomic identity of nonparasitic lampreys in Japan and Sakhalin Island, Russia with low trunk myomere counts’, page 42). Salewski (2003) argued that the worldwide trend of satellite species in lampreys perhaps represented cases of sympatric speciation. Although lamprey species are usually either parasitic or nonparasitic, *Le. appendix* is the only nonparasitic species within the genus that exhibits very rarely what has been termed facultative parasitism (Docker 2009; Renaud 2011; Renaud and Cochran 2019). Eight “giant” *Le. appendix* adults (260–354 mm in total length) have been reported from Lake Huron and Lake Michigan basins (Manion and Purvis 1971; Vladykov and Kott 1980; Cochran 2008). Because these adults exceed the maximum total length of 240 mm reported for the larvae of the species (Mundahl et al. 2005) they must have fed post-metamorphosis. The retained capacity of some individuals to feed post-metamorphosis argues for a recent divergence of *Le. appendix* from *Le. camtschaticum* (Renaud 2011; Renaud and Cochran 2019).

**Table 8.** Trunk myomeres, dentition, oral papillae, and oral fimbriae in adult types of *Lampetra mitsukurii* Hatta, 1901 and *Lethenteron matsubarai* Vladykov & Kott, 1978. Numbers in parentheses are frequencies of character states. Abbreviation: u, unicuspid.

	*Lampetra mitsukurii*	*Lethenteron matsubarai*
	ZMB 20638 (2 syntypes)	CMNFI 1984-274 (2 paratypes)
Type locality	Japan	Shokotsu River, Hokkaido Island, Japan
Trunk myomeres	66–67	69–70
Supraoral lamina	1u–1u (2)	1u–1u (2)
Endolateral formula	2–2–2 (4)	2–2–2 (3), 2–2–1
Infraoral lamina	6u (2)	6u (2)
Rows of anterials	1, 2	2 (2)
Rows of exolaterals	0 (4)^1^	0 (4)
Rows of posterials	1, 2^2^	1 (2)
First anterial row	4u, 5u	3u, 4u
First posterial row	20u, 21u	18u, 19u
Transverse lingual lamina	undetermined (2)	2u–I–4u, 3u–I–3u
Longitudinal lingual laminae	undetermined (2)	4u, undetermined
Oral papillae	11, 23	19, 20
Oral fimbriae	87, 106	undetermined (2)

^1^ One exolateral tooth on the left side and two on the right side in one specimen. ^2^ 22 unicuspid teeth are present in the second row of posterials.

### *Lethenteron* species with exolaterals

A number of individuals of various *Lethenteron* species possess exolaterals [see Kott (1974) in reference to *Le. appendix* (appeared as *Lethenteron lamottei*), in which 21.1% of individuals possessed one or two exolaterals in one or both exolateral fields; Vladykov and Kott (1978a) in reference to *Le. alaskense*, in which the holotype possesses one exolateral in each one of the exolateral fields and one paratype possesses on both exolateral fields a complete exolateral row, which they refer to as a supplementary marginal row; Renaud and Naseka (2015) in reference to one individual each of *Le. reissneri* and *Le. camtschaticum*, in which they respectively possessed two and one exolateral teeth on one of the fields]. In this study, we recorded one or two exolaterals per exolateral field in two individuals of *Le. camtschaticum* (one from the Kamchatka River and one from the Tobol River), one individual of *Le. kessleri* from the Ob’ River and in one syntype of *Le. mitsukurii* (Tables 5, 8).

**Table 9.** Shape and pigmentary characters in adult types of *Lampetra mitsukurii* Hatta, 1901 and *Lethenteron matsubarai* Vladykov & Kott, 1978. Numbers in parentheses are frequencies of character states. Pigmentation coverage as follows: -, absent to < 1%.

	*Lampetra mitsukurii*	*Lethenteron matsubarai*
	ZMB 20638 (2 syntypes)	CMNFI 1984-274 (2 paratypes)
Type locality	Japan	Shokotsu River, Hokkaido Island, Japan
Caudal fin shape	spade-like (2)	spade-like, undetermined
**Pigmentation**		
Caudal fin	- (2)	- (2)
Second dorsal fin	no blotch, undetermined	no blotch (2)
Lateral line neuromasts	unpigmented (2)	unpigmented (2)
Gular	undetermined (2)	- (2)

### *Petromyzon japonicus*, a synonym of *P. marinus camtschaticus*

The original description of *P. marinus camtschaticus* based on a single adult (now lost) is very different from that of *P. japonicus* represented by two extant adult syntypes, one of which was selected as the neotype of the former taxon by Kottelat (1997) thereby making it a lectotype of the latter taxon. The original description of *P. marinus camtschaticus* refers to a supraoral lamina bearing two bicuspid teeth separated by a wide bridge, two pairs of bicuspid endolaterals and no row of posterial teeth is mentioned or depicted, while in that of *P. japonicus* (Fig. 2) the supraoral lamina has two unicuspid teeth separated by a wide bridge, three pairs of bicuspid endolaterals (although the accompanying drawing shows 1–2–2 on the left and 2–2–1–2 on the right side) and a single row of posterials. However, both taxa exceed 300 mm TL indicating that they represent parasitic species and possess a u-shaped transverse lingual lamina with an enlarged median cusp. The number of teeth on the transverse lingual lamina in the original description of *P. m. camtschaticus* is seven and nine in that of *P. japonicus* although in the type material from Japan we counted 2u–I–6u and 2u–I–3u (Table 2). The disc length of 5.2% TL estimated from the original description of *P. m. camtschaticus* is similar to the disc length of 4.1–4.8% TL for the type material from Japan (Table 1). The original description of *P. m. camtschaticus* reports seven unicuspid teeth on the infraoral lamina and that of *P. japonicus* reports six unicuspid teeth (although we counted seven in the accompanying drawing). However, the type material from Japan exhibits counts of 1b4u1b and 1b5u (Table 2). The second dorsal fin is stated to be blackish in the original description of *P. m. camtschaticus* and although no mention is made of its condition in the original description of *P. japonicus* both types from Japan exhibited a blotch on the second dorsal fin (Table 3). We agree with the action by Kottelat (1997) of making *P. japonicus* a synonym of *P. marinus camtschaticus* and suggest that the specimen described by Tilesius (1811) is atypical in the condition of its supraoral lamina and number of bicuspid endolaterals, while the absence of a row of posterials is an oversight on his part as these teeth are relatively small in comparison to the others that he did describe.

### Identity of the lamprey in Steller’s unpublished manuscript

Because Tilesius (1811) had one lamprey specimen only, he added the unpublished description (Indice Piscium Camtschaticorum, manuscript F) by Georg Wilhelm Steller (1709–1746) of a metamorphosed lamprey collected near the mouth of Bolschaja River brought to the latter on 18 June 1738 (Julian calendar; 29 June 1738 Gregorian calendar). However, Steller left St. Petersburg in January 1738 to join the Second Kamchatka Expedition (1733–1743) of Vitus Jonassen Bering (1681–1741), and by January 1739, had only reached Yeniseysk (on the Yenisei River), where he met Johann Georg Gmelin (1709–1755); still over 3,900 km from the Kamchatka Peninsula. Although there is a Bolshaya River on the western coast of Kamchatka, it is, therefore, improbable that this lamprey was obtained from there. It is more probable that the lamprey came from the Yenisei River, the name being derived from Ionessi (= Yonessi) meaning Bolschaja (= Bolshaya) voda or Big water in Evenki, the language spoken by the local Evenks people. This 333 mm TL lamprey adult is described as having variegated (mottled) body coloration with a brown dorsal surface and brown lines extending down in a wavy pattern into the shiny green-bronze sides of the body while the type of *P. m. camtschaticus* is not mottled. Otherwise, the characters described are similar to those of *P. m. camtschaticus*: body anguilliform; two dorsal fins, interspace between them ca. seven mm; posterior (second) dorsal fin blackish at the top; teeth yellowish; supraoral lamina with two teeth; infraoral lamina with six teeth. Additional characters described are: snout length is ca. 25 mm (≈ 7.5% TL); eye length is ca. 2.5 mm (≈ 0.8% TL); iris bronze colored. The common name Kanaháisch of this variegated lamprey is almost identical (Canaháisch) to the one reported for *P. m. camtchaticus* by Tilesius (1811). Although both *Le. camtschaticum* and *Le. kessleri* occur in the Yenisei River (see below ‘Sympatric parasitic and nonparasitic taxa’, page 40), the lamprey in Steller’s manuscript is *Le. camtschaticum* based on its size (333 mm TL). The only other study that reports a mottled body coloration in metamorphosed lampreys from Russia is that of Berg (1931) based on four non-type adults of *La. j. septentrionalis* [= *Le. camtschaticum*; see below ‘*Lampetra japonica septentrionalis* a synonym of *Petromyzon japonicus*’, page 26] measuring 279–345 mm TL from the Onega River.

### *Lampetra japonica septentrionalis* not derived from *La. fluviatilis*

We reject the hypothesis proposed by Ioganzen (1935a) that the similar dentition of *La. j. septentrionalis* and *La. j. japonica* is not indicative of their common origin and that *La. j. septentrionalis* is instead derived from *La. fluviatilis* and should therefore be called *La. fluviatilis septentrionalis*. Ioganzen (1935a) argued that the differences in dentition in *La. j. septentrionalis* versus *La. fluviatilis* in the second endolateral tooth (bicuspid versus tricuspid, exceptionally quadricuspid, respectively) and posterial row (present versus absent, respectively) were not stable taxonomic characters to distinguish the two. However, Ioganzen (1935a) provided no evidence for the presence of a tricuspid or quadricuspid second endolateral tooth in *La. j. septentrionalis* and merely suggested that this was due to insufficient samples. Berg (1948) hypothesized that the absence of lower labials (i.e., posterials) in three of 19 adults of *La. japonica* from the Vyg River, White Sea basin, could be explained through hybridization of this species with *La. fluviatilis* in the post-Tertiary period during which the White and Baltic sea basins were in communication. Unfortunately, we did not examine this particular material, although the single adult we did examine from the Vyg River (Table 5, ZIN 8545) possessed a row of posterials. Given that all lamprey individuals that we examined and were stated by Berg (1931) to be without posterials in fact possessed them (this study; Renaud and Naseka 2015), we believe that the hybridization explanation does not need to be invoked. Additionally, the cladistic analysis of Gill et al. (2003) based on morphological characters that included dentition showed that *La. fluviatilis* was not sister to *Le. camtschaticum*.

### *Lampetra japonica septentrionalis*, a synonym of *Petromyzon japonicus*

Berg (1931) distinguished *La. j. septentrionalis* from *La. j. japonica* by the smaller length of its upstream migrants (227–430 mm TL versus 352–625 mm TL, respectively) and its lower fecundity (24,086–25,144 eggs versus 80,825–107,015 eggs, respectively). Although Berg (1948) did not explain why he synonymized *La. j. septentrionalis* with *La. j. japonica*, he stated that the upstream migrating lampreys from the Mezen’ River, White Sea basin, were not smaller than those of the Pacific Ocean basin [540 mm TL (Manteyfel’ 1945) versus 625 mm TL (Berg 1931), respectively], and hence, one of his two diagnostic characters no longer held. Furthermore, Berg (1948) stated that the average number of eggs within lamprey from the Taui River, Okhotsk Sea basin, was ca. 40,000. This is intermediate between the 24,086–25,144 eggs for *La. j. septentrionalis* and 80,825–107,015 eggs for *La. j. japonica* reported in Berg (1931), thereby weakening the strength of his second diagnostic character. Additionally, Morozova (1956) recorded fecundities ranging from 50,000 to 124,000 eggs in *La. japonica* measuring 335–481 mm TL from the Amur River near Elabuga and Malmyzh, Russia, Yamazaki et al. (2001a) recorded fecundities ranging from 62,936–119,180 eggs in *Le. japonicum* measuring 363–442 mm TL from Hokkaido and Honshu islands, Japan, and Nursall and Buchwald (1972) recorded fecundities ranging from 9,790 to 29,780 eggs in 18 mature *Le. japonicum* females (TL not stated) from Great Slave Lake, Slave River, and Hay River, Northwest Territories, Canada. Our comparison of morphometrics, trunk myomeres, dentition, oral papillae and fimbriae, caudal fin shape and pigmentary characters of the type material of *Petromyzon marinus camtschaticus*, *P. japonicus* and *La. j. septentrionalis* (Tables 1–3) did not reveal any diagnostic differences between these three nominal taxa and we therefore consider them to be synonyms. Makhrov and Lajus (2018) suggest that *Le. camtschaticum* originated in the Pacific Ocean basin and colonized the Eurasian Arctic Ocean basin postglacially because in the median joining network analysis of mtDNA haplotypes, the Northern European haplotypes occur at the end of branches indicating their recent origin. Furthermore, on the eastern portion of its distribution, Walters (1955) proposes that *Le. camtschaticum* (appeared as *La. japonica*) colonized Arctic Canada through two possible routes: Arctic Alaska and the Yukon Valley; and that this could have occurred during the presence of the Bering Strait land bridge linking Eurasia and North America. Walters (1955) further states that *Le. camtschaticum* can also disperse across marine waters, and therefore, this is another possible route for its colonization of Arctic Canada. Yamazaki et al. (2014) provide evidence based on their study of seven polymorphic nuclear microsatellite loci in 12 anadromous *Le. camtschaticum* populations distributed between Velikaya River, Chukotka, Russia in the north and Jinzu River, Honshu Island, Japan in the south, that the species has considerable marine dispersal ability and low homing ability. There is a caveat, however, associated with that study. Only three localities on Honshu and Hokkaido islands, Japan out of the 12 contained adults; the remaining samples were ammocoetes which are notoriously difficult to identify to species and were not dealt with in the present study.

### *Petromyzon ernstii*, a synonym of *Lethenteron camtschaticum*

*Petromyzon ernstii* is considered a junior synonym of *Le. camtschaticum* on the basis of its adult total length of 310 mm (Dybowski 1872a). The adult size attained by *P. ernstii* indicates that it feeds post metamorphosis and *Le. camtschaticum* is the only species to do so in the genus. The dentition of *P. ernstii* is not fully described by Dybowski (1872a), but what is described generally agrees with that of the neotype of *P. m. camtschaticus* (Table 2): supraoral lamina with two strong cusps; three endolaterals on each side with the top two endolaterals bicuspid (the type of the bottom endolateral is not given); infraoral lamina with two bicuspid teeth laterally and an average of four unicuspid teeth internally; two rows of anterials; one row of posterials. While the counts of 19 teeth on the transverse lingual lamina and 12 on each of the longitudinal lingual laminae reported by Dybowski (1872a) are respectively lower (9) and undetermined in the neotype of *P. m. camtschaticus* (Table 2), in the types of the latter’s junior synonyms, *P. japonicus* and *La. j. septentrionalis*, the ranges observed are much closer to reaching those values (respectively, 6–16 and seven or eight; Table 2). If we include topotypes of *La. j. septentrionalis* the counts are even closer or encompassing (respectively, 12–17 and 9–13; Table 2). Notwithstanding the fact that we did not observe a count of 19 teeth on the transverse lingual lamina, we found a count of 18 in a specimen from the Tobol River identified as *La. j. septentrionalis* by Berg (1931) (Table 5), and therefore, we believe that *P. ernstii* is a junior synonym of *Le. camtschaticum*.

### Variability in the infraoral lamina dentition of *Le. camtschaticum*

Berg (1948) reported that *La. j. japonica* + *La. j. septentrionalis* (= *Le. camtschaticum*) usually had six mandibular (infraoral) teeth, occasionally seven (exceptionally nine in the Kamchatka River). We found six in the types (Table 2) and six to eight in the non-types (Table 5). According to Kucheryavyi et al. (2007a), the infraoral lamina in typically anadromous *Le. camtschaticum* from the Utkholok River drainage, Kamchatka possessed one bicuspid tooth on either side, and three to seven unicuspid teeth internally, giving a total of five to nine teeth. Kucheryavyi et al. (2007a) and Nazarov et al. (2011) for the Kol’ River drainage, Kamchatka, also reported that rarely one of the lateralmost teeth was unicuspid. We also found a lateralmost unicuspid tooth in the paralectotype of *Petromyzon japonicus* (ZMB 6476; Table 2) and one topotype of *La. j. septentrionalis* (ZIN uncat.; Table 2).

### Real or perceived distributional discontinuity between the populations of parasitic *Le. camtschaticum* in Russia?

In regard to the wide discontinuity in the distributions of the populations of parasitic *Le. camtschaticum* reported by Berg (1931) as *La. j. septentrionalis* and *La. j. japonica* and later (Berg 1948) combined as *La. j. japonica*, this study has re-identified as *Le. camtschaticum* some of the samples in the intervening Siberian waters and considered by the aforementioned author as well as Ioganzen (1935b) as nonparasitic *La. j. kessleri*, thereby narrowing the gap between the distributions. Firstly, Berg (1931, 1948) tentatively considered and Ioganzen (1935b) undoubtedly considered *P. dentex* Anikin, 1905 to be a junior synonym of *La. j. kessleri*. However, we suggest instead that it is a junior synonym of *Le. camtschaticum* because Anikin (1905) stated that the teeth of the two approximately 160 mm TL specimens collected at the mouth of the Yenisei River are brown (indicating strong keratinization) instead of dark yellow as they usually are in *P. kessleri* and that although they have the same number and arrangement of teeth as in *P. kessleri*, these are larger and sharp, resembling those of *Petromyzon* (= *Lampetra*) *fluviatilis* rather than *P.* (= *L.*) *planeri*. Furthermore, Egorov (1985) reports the presence of *La. j. kessleri* adults up to 322 mm TL from the Yenisei Gulf, which on the basis of size we identify as *Le. camtschaticum*. Therefore, in our opinion, the range of *Le. camtschaticum* extends further east than the Ob’ River drainage to the Yenisei River. Secondly, six specimens from the Anadyr Liman (= Estuary) are also re-identified as *Le. camtschaticum* rather than *Le. kessleri*. Anikin (1905) stated that *La. j. kessleri* (appeared as *P. kessleri*) reached an adult TL of 210 mm, but Berg (1931) stated that it reached, albeit rarely, 220–260 mm. Although we acknowledge that a TL of 221 mm can be attained (Table 4, ZIN 6174; only 212 mm when re-measured by us likely caused by shrinkage over time), we suggest that the 258 mm TL spent female [ZIN 23154 in Berg (1931)] is a *Le. camtschaticum* rather than *La. j. kessleri* because it was collected in brackish water of the Anadyr Estuary and was attached to a dog salmon, also called chum salmon (*Onchorhynchus keta*). Furthermore, it exceeds by 38 mm the longest ammocoete reported by Berg (1931) for *La. j. kessleri* (ZIN 6306, 220 mm TL from the Irtysh River at Omsk) even without taking into account the shrinkage in body length that occurs in a nonparasitic species between the beginning of metamorphosis and spawning. The two adults 135–138 mm TL (ZIN 23158) with very sharp teeth identified as *La. j. kessleri* by Berg (1931) are also re-identified as *Le. camtschaticum* because they were also collected in brackish water and attached to a chum salmon. The adult 144 mm TL (ZIN 14371) identified as *La. j. kessleri* by Berg (1931) is also re-identified as *Le. camtschaticum* (Tables 4–6) because it possesses the silvery body coloration of an anadromous downstream migrant. Later, Berg (1948) suggested that what he called *La. j. japonica* is possibly present in the Anadyr River based on a 260 mm specimen with sharp teeth collected in its estuary on 7 August 1938. Two other specimens with sharp teeth and thick intestines from the Anadyr Estuary identified by Berg (1948) as *La. j. kessleri* are re-identified as *Le. camtschaticum*; one (155 mm TL) was attached to a sculpin (Cottidae) and the other (144 mm TL) to a chum salmon. Chereshnev (2008) also reports the presence of anadromous *Le. camtschaticum* in the Anadyr River from the estuary to ca. 600 km upstream. Therefore, we believe that *Le. camtschaticum* occurs in the Anadyr River and this extends its range northward from Kamchatka. Between the Yenisei and Anadyr River drainages are only two major rivers: the Lena and Kolyma; both within the region of Yakutia, also known as Republic of Sakha. According to Kirillov and Chereshnev (2006) and Kirillov et al. (2014), only nonparasitic *Lethenteron* lampreys occur in this entire region from the Anabar (west of the Lena) to the Kolyma rivers. However, these authors provide very little information about the lampreys and two things suggest that, at least in the case of the Kolyma River, anadromous, parasitic lamprey may be present. According to Kirillov et al. (2014) the mature adults at the beginning of the spawning season attain 240 mm TL, which slightly exceeds the maximum total length of 230 mm attained by *Le. kessleri* (see below), and lamprey are known down to the estuary, which would not be expected in a brook lamprey. Further investigations are required to confirm this.

### Adult total length attained by *Le. kessleri*

Although Berg (1931) considered *La. j. kessleri* to be a small non-migratory (i.e., resident) form in which the larvae at metamorphosis were larger than the adults, the material at his disposal did not support this contention. Berg (1931) stated that the longest ammocoete was 220 mm and adults reached 220–260 mm. We have explained above that *Le. kessleri* adults reach 221 mm TL, but have no direct evidence that they exceed this value. However, Poltorykhina (1971, 1974) conducted the most extensive studies of *Le. kessleri* examining 50 ammocoetes, 104 metamorphosing ammocoetes and 100 adults in the first study and 300 adults in the second study collected from the upper Irtysh River system (Ob’ River drainage) and reports that 218 mm is the mean TL attained by 50 ammocoetes, 233 mm is the mean TL attained by 12 metamorphosing ammocoetes at the penultimate stage of metamorphosis, and 230 mm is the maximum TL attained by adults. According to Poltorykhina (1971) the apparent increase in length between ammocoetes and adults is not the result of parasitic feeding post metamorphosis, but due to continued feeding of metamorphosing ammocoetes, at first on detritus and unicellular algae and later exclusively on unicellular algae, even though the oral cirri are lost and replaced by the rudiments of teeth over the course of metamorphosis. The latter is perhaps achieved through grazing of unicellular algae on rocks via the action of the lingual laminae and piston cartilage as suggested by Renaud (2011). Although Poltorykhina (1971) did not comment on whether or not the digestive tract remained open throughout metamorphosis, one must assume that it is patent at least for most of the period because growth occurred and algae were found in the intestine up until and including the last stage of metamorphosis. According to Poltorykhina (1971, 1974) the adults possess 67–72 trunk myomeres, which overlaps the range based on our examination of *Le. kessleri* type material (70–74; Table 2).

### Variability in the dentition of *Le. kessleri*

Ioganzen (1935b) reported that *La. j. kessleri* from the Ob’ River drainage usually has two teeth on the supraoral lamina (n = 15) and exceptionally four (n = 1). However, his drawing (1935b: fig. 7) of the supraoral lamina in the latter specimen from the Ob’ River near Bogorodskoye is interpreted as two bicuspid teeth instead of four unicuspid teeth and Egorov (1985) is also in agreement with this interpretation. However, Zhuravlev and Lomakin (2017) reported the case and provided a photograph of four unicuspid teeth on the supraoral lamina, one at each end and two on the bridge, in one of 54 individuals of *Le. kessleri* from the Belokurikha River, upper Ob’ drainage (53 individuals had only one tooth at each end). In her extensive study of *Le. kessleri* from the upper Irtysh River system (Ob’ River drainage), Poltorykhina (1974) reported usually two teeth on the supraoral lamina (n = 297) and three cases in which an additional tooth was found on the bridge. We also found a case of three teeth on the supraoral lamina (ZIN 6310, Table 5). Poltorykhina (1974) stated that the infraoral lamina possesses five to ten teeth, usually six or seven, with the lateralmost being bicuspid and in 16 of 300 individuals some of the internal ones are also bicuspid. In five syntypes of *P. kessleri* (Table 2) we found six or seven teeth, with the lateralmost bicuspid and in one individual from the Yenisei River (ZIN 14441, Table 5) we recorded five teeth with the lateralmost bicuspid. Ioganzen (1935b) reported four bicuspid endolaterals on either side of the oral disc in a syntype of *P. kessleri* from the Tom’ River (Ioganzen 1935b: table 3, TGU 3700). We did not find this specimen during AMN’s visit to TGU in 2011. All of the syntypes that we examined (Table 2) had three endolaterals only on either side, most of which were bicuspid (n = 9) and one with the formula 2–2–3. Additionally, Anikin (1905) only reported three bicuspid endolaterals in the original description of *P. kessleri*. Poltorykhina (1974) also only recorded three endolaterals on either side of the oral disc (n = 300), and while the majority were bicuspid, in 36 individuals the formula was 2–2–1 and in 16 it was 2–1–2. Nazarov (2012) stated that usually there were three, but sometimes only two endolaterals on one side in material from the middle Yenisei River drainage. Nazarov (2012) reported three to five teeth in the first anterial row in individuals from the middle Yenisei River drainage, while we recorded invariably five in five syntypes of *P. kessleri* (Table 2) and two to six in non-types of *Le. kessleri* (Table 5). Poltorykhina (1974) reported that the lower circumoral teeth (= first posterial row) consist of a single row with 16–25 teeth and an unspecified number of individuals do not possess any. We recorded 24–29 teeth in three syntypes of *P. kessleri* (Table 2) and 18–31 in non-types of *Le. kessleri* (Table 5).

### *Lethenteron kessleri* without posterials?

The presence of well-developed lower labial teeth (= posterial row) in *Le. kessleri* versus weakly developed or completely absent lower labial teeth in *Le. reissneri* was the diagnostic character used by Berg (1931, 1948) to distinguish the two taxa. However, Renaud and Naseka (2015) showed that a complete and well-developed posterial row was usually present in *Le. reissneri*. Berg (1931) reported “a very aberrant specimen” of *Le. kessleri* from the upper Yenisei River (ZIN 14441) without teeth on the lower lip (i.e., no posterials) and Berg (1948) suggested that it was possibly an aberrant *Le. kessleri* resembling *Le. reissneri*. However, we re-examined this specimen and found one row of posterials with 18 unicuspid teeth (Table 5). This situation is reminiscent of the case of three specimens identified by Berg (1931) as *Le. reissneri* without posterials, but found by Renaud and Naseka (2015) to possess them. While one of the three specimens was determined by Renaud and Naseka (2015) to be *Le. reissneri* (Shangshi River, People’s Republic of China), they re-identified the other two as *Le. camtschaticum* (Samarga and Sedanka rivers, Russia).

We suggest that the ten syntypes of *P. kessleri* retrieved in relatively good condition from the digestive tract of a Common gull [TGU 9 = TGU 3696 in Ioganzen 1935b: table 3] on 28 June 1903 were spawning because lampreys congregate in shallow waters during that time making them susceptible to bird predation. This was also the contention of Holčík (1986b). Additionally, Ioganzen (1935b) stated that the two dorsal fins touched in all ten individuals indicating that they were mature adults.

### Identity of lampreys in the Utkholok River drainage, Kamchatka

Kucheryavyi et al. (2007a) reported the presence of three forms of *Lethenteron camtschaticum* in the Utkholok River drainage, Kamchatka, Russia; typically anadromous, anadromous forma praecox, and resident. All three forms spawned together in June 2005 and possessed a dark blotch at the apex of their second dorsal fin and well pigmented spade-like caudal fins. The total lengths of the three forms were: 174–350 mm (typically anadromous, mature individuals of both sexes combined), 145–220 mm (anadromous forma praecox, mature individuals of both sexes combined), and 100–165 mm (resident, mature individuals of both sexes combined). Kucheryavyy (2014) reported anadromous adults of *Le. camtschaticum* on the Kamchatka Peninsula up to 452 mm TL. The supraoral lamina in the three forms possessed one unicuspid tooth on either end and in the case of the typically anadromous form rarely one bicuspid tooth on either end, as was also the case in the specimen of *P. m. camtschaticus* in Tilesius (1811: pl. IX, fig. II). The endolaterals in the three forms generally consisted of three bicuspid teeth on either side. Rarely, in the case of the typically anadromous form, the lower left or right endolateral was unicuspid or there were four bicuspid endolaterals or there were three bicuspid with one unicuspid tooth at the lower position, and rarely in the case of the resident form, the lower endolateral was unicuspid or there were four bicuspid endolaterals. The infraoral lamina in the three forms generally possessed one bicuspid tooth on either side, and respectively, three to seven, four or five, and four to six unicuspid teeth internally. Rarely, in the case of the typically anadromous form, one of the lateralmost teeth was unicuspid, and in the case of the resident form, one or both lateralmost teeth were unicuspid. The number of posterials for the three forms was respectively, 13–28, 12–22, and 12–25. These values are lower than the combined values of 18–29 posterials, based on our examination of type or topotype material of *P. m. camtschaticus*, *P. japonicus*, *P. kessleri*, and *La. j. septentrionalis* (Table 2), but may reflect the larger sample sizes examined by Kucheryavyi et al. (2007a). Remarkably, the range in trunk myomeres for the three forms was extremely broad for a small river drainage being respectively 55–79, 57–71, and 57–78. The lower end counts may at least partly be explained by the method of counting which was based on a vertical line drawn in front of the cloaca rather than using the lower angle of the posterior myoseptum lying at or anterior to the anterior edge of the cloaca as in Renaud (2011). We interpret the typically anadromous form as being *Le. camtschaticum* and both the anadromous forma praecox and the resident form as being *Le. kessleri*. The resident form is stated by Kucheryavyi et al. (2007a) as not feeding after metamorphosis. The reasons we identify the anadromous forma praecox as *Le. kessleri* is because there is no clear evidence that it is anadromous or feeding as an adult as the intestinal contents of 16 individuals were examined by Kucheryavyi et al. (2007a), and while five did not contain any food, six contained small algae and five contained brown material similar in color to the food of ammocoetes, and furthermore, their adult size range (145–220 mm TL) falls under the maximum 230 mm TL reported for adults of the species by Poltorykhina (1974). If the anadromous forma praecox had spent several months to a year in the sea, as Kucheryavyi et al. (2007a) contend, one would not expect any remnants of larval food in their intestine. While we identify as *Le. camtschaticum* four small adults collected in the Kamchatka River and measuring 154.5–198 mm TL (Table 4: ZIN 23440, 23441, 23590), these individuals possessed intestinal diameters 2.5–4.5 mm (Table 4) and were therefore at the beginning of their adult life unlike the spawning individuals in the Utkholok River drainage presumed by Kucheryavyi et al (2007a) to be anadromous forma praecox*Le. camtschaticum*. In fact, Berg (1931) stated that three of the four individuals collected June 1908 (ZIN 23441) and 7 July 1909 (ZIN 23590) measuring a combined 154.5–198 mm TL were descending towards the ocean. The fecundity of the typically anadromous form was 12,272–34,586 eggs (Kucheryavyi et al. 2007a), which overlaps the lower end of the fecundity (24,086–107,015 eggs) recorded by Berg (1931, 1948) for *Le. camtschaticum*. Kucheryavyi et al. (2007a) did not record the fecundity for the anadromous forma praecox, but stated that the fecundity of the resident form was 468–3,441 eggs. This we take to be the fecundity for *Le. kessleri*. It broadly overlaps with the fecundity reported by Poltorykhina (1973) [cited in Holčík (1986b) and Makhrov et al. (2013)] for *Le. kessleri* (i.e., 1,820–5,800 eggs) from the upper Irtysh River system, Ob’ River drainage, as well as the fecundity reported by Zhuravlev and Lomakin (2017) for *Le. kessleri* (i.e., 465–1,350 eggs) from the Belokurikha River, Ob’ River drainage, and slightly overlaps with the fecundity reported by Karasev (2008) for *Le. kessleri* in the lower Tobol River (i.e., 3,161–7,208 eggs), also in the Ob’ River drainage, and is very close to the fecundity reported by Kuderskiy and Mel’nikova (1970) for *Le. kessleri* (i.e., 651–3,096 eggs) in the Yemtsa River (Severnaya Dvina River drainage), and to that of *Le. alaskense* provisionally suggested here as a synonym of *Le. kessleri* (see below ‘Taxonomic key to adults of *Lethenteron*’, page 53). The fecundity of *Le. alaskense* from Brooks River, Alaska, U.S.A. and Martin River, Northwest Territories, Canada was reported by Vladykov and Kott (1978a) as 2,188–3,477 eggs. It is also very similar to the fecundity recorded by Karasev (1987) for *Le. reissneri* from the Ingoda River, Russia (1,720–3,360 eggs). The extremely low fecundity of 117 eggs recorded by Novikov (1966), in a 134 mm TL*Le. kessleri* retrieved from the stomach of a Northern pike, *Esox lucius*, collected in July 1963 in the Kolyma River, Russia, may have been a partially spent female.

### Identity of lampreys in the Kol’ River drainage, Kamchatka

Similar to the case reported above in the Utkholok River drainage, Nazarov et al. (2011) stated the presence of three forms of *Le. camtschaticum* in the Kol’ River drainage, Kamchatka, Russia; typically anadromous (243–297 mm TL, mature individuals sexes combined), anadromous forma praecox (190.5–237 mm TL, two spent females only), and resident (110–141 mm TL, mature individuals sexes combined). However, whereas all three forms are reported to possess a blotch on the second dorsal fin (their table 2), only the typically anadromous and anadromous forma praecox possess one (their fig. 5a, b, respectively), whereas the resident form appears to possess none (their fig. 5c depicting a male and a female). Additionally, the typically anadromous and anadromous forma praecox possess a strongly pigmented caudal fin (their fig. 5a and 5b, respectively), whereas the resident form appears to possess no caudal fin pigmentation (their fig. 5c). The two anadromous forms are said to be parasitic and the resident form nonparasitic. The supraoral lamina in the three forms possessed one unicuspid tooth on either end. The endolaterals in the three forms generally consisted of three bicuspid teeth on either side. Rarely, in the case of the typically anadromous form, and sometimes, in the case of the resident form, the lower left or right endolateral was unicuspid. The infraoral lamina in the three forms generally possessed one bicuspid tooth on either side, and respectively, four to seven, five or six, and five or six unicuspid teeth internally. Rarely, in the case of the typically anadromous form, one of the lateralmost teeth was unicuspid. The number of posterials for the three forms was respectively, 13–20, 17–22, and 15–21. The range in trunk myomeres for the three forms was respectively, 71–76, 73–75, and 67–74. The fecundity of the typically anadromous form was 24,038–31,050 eggs, which virtually completely overlaps with the lower end of the fecundity (24,086–107,015 eggs) recorded by Berg (1931, 1948) for *Le. camtschaticum*. Nazarov et al. (2011) did not record the fecundity for the anadromous forma praecox, as the only two females were spent, but stated that the fecundity of the resident form was ca. 3,200 eggs. Nazarov et al. (2011) reported that resident lamprey either spawned communally with typically anadromous and anadromous forma praecox or independently. This is reminiscent of the situation reported by Savvaitova and Maksimov (1978) of communal spawning between large (230–320 mm TL) and small (100–140 mm TL) lampreys and independent spawning of small lamprey in the Levyy Kolkalvayam River, Utkholok River drainage. We interpret the typically anadromous and anadromous forma praecox as being *Le. camtschaticum* and the resident form as very similar to *Le. mitsukurii* in terms of the absence of a blotch on the second dorsal fin and the unpigmented caudal fin, but different in its possession of bicuspid rather than unicuspid lateralmost teeth on the infraoral lamina (see below ‘*Lethenteron mitsukurii* distinct from *Le. kessleri* and *Le. reissneri*’, page 37). We, therefore, defer judgment on the identity of the resident form until more information becomes available. However, it is significant that Vladykov and Kott (1979c) reported the presence of *Le. matsubarai* on the Kamchatka Peninsula (see below ‘*Lethenteron matsubarai*, a synonym of *Le. mitsukurii*’, page 38). Khusainova and Karpenko (2017) confirmed the presence of *Le. camtschaticum* in the Kol’ River based on prespawning individuals measuring 312–351 mm TL.

### Identity of lampreys in the Lake Azabach’e basin, Kamchatka

Karpenko et al. (2013) described 37 spawning individuals of *Lethenteron* measuring 147.1–172.0 mm TL and collected on 12 July 2012 from Dyakonovskiy Creek, Lake Azabach’e basin, but they could not identify them to species. It would appear that the individuals were partially spent because the four females examined possessed only 16–126 eggs and in the two in which the eggs were measured they were ca. 1.0 mm in length. According to Karpenko et al. (2013) these adults possess two or three teeth on the supraoral lamina (usually one unicuspid tooth at each end, but in one case the tooth at one end is bicuspid, and in another case an additional unicuspid tooth is present on the bridge between the unicuspid lateral teeth), three bicuspid endolaterals on each side, the infraoral lamina usually with two lateralmost teeth bicuspid and four or five internal teeth unicuspid (sometimes the two lateralmost as well as the four, five or six internal teeth are unicuspid), one row of small posterials, and 66–74 trunk myomeres. While the characters of dentition and trunk myomeres all agree with *Lethenteron*, the small total length of the spawning individuals would indicate a nonparasitic species; either *Le. reissneri* or *Le. kessleri*. However, the diagnostic characters distinguishing these two species (i.e., second dorsal fin pigmentation and transverse lingual lamina dentition; see below ‘*Lethenteron kessleri* distinct from *Le. reissneri*’, page 35) were not recorded, and therefore, we cannot determine the species’ identity. Bugayev et al. (2007) had reported the presence of *Le. reissneri* in Lake Azabach’e, but we have re-identified the 185 mm TL adult as *Le. camtschaticum* because its intestine contained half-digested fish flesh. Lake Azabach’e belongs to the Kamchatka River drainage, and the presence of *Le. camtschaticum* is well documented in this river drainage (Berg 1931, 1948; Bugayev et al. 2007). Khusainova and Karpenko (2017) collected additional spawning individuals from Dyakonovskiy Creek in 2014 (n = 145) and compared them with prespawning material from the Kol’ River (western Kamchatka) collected in 2013 (n = 19). These authors concluded based on total length that the lamprey from the Dyakonovskiy Creek measuring 132–190 mm TL is *Le. reissneri* and the one from the Kol’ River measuring 312–351 mm TL is *Le. camtschaticum*. While we agree with the identification of *Le. camtschaticum* for the Kol’ River material, we believe that the identification of the material from Dyakonovskiy Creek cannot be established because, as in Karpenko et al. (2013), neither the second dorsal fin pigmentation nor the transverse lingual lamina dentition was examined by Khusainova and Karpenko (2017).

### *Ammocoetes aureus*, a synonym of *Le. camtschaticum*

Despite having been placed in the genus *Ammocoetes*, *A. aureus* is clearly based on an adult individual. The holotype measures 381 mm TL, and it possesses supraoral and infraoral laminae and eyes. Even though the original description is fragmentary, the size of the adult individual, the condition of its supraoral (two cusps) and infraoral (seven cusps, the lateralmost one on either end enlarged) laminae indicate that *A. aureus* is a junior synonym of *Le. camtschaticum*. Nelson (1887) reports that *A. aureus* (= *Le. camtschaticum*) ascends the Yukon River, Alaska, at least up to Nulato and that the native Alaskan name for the Arctic lamprey is Nû-mug-û-shûk. He further reports that the upstream-migrating lamprey passed through Anvik in the evening of 26 Nov. 1879 and that the native Alaskans catch them through holes in the ice with sticks having two short cross bars at the lower end or dipnets and extract their oil for eating and as a substitute for seal oil in lamps. On the other hand, Turner (1886) reports that *A. aureus* (= *Le. camtschaticum*) ascends the Yukon River in the latter part of December, reaches the Russian Mission – Anvik river section by the middle of February and by the latter part of April, Fort Yukon, Alaska, over 1,600 km upriver of the mouth. Turner (1886) also states that the spawning run passing through a given locality lasts approximately three weeks.

### *Lethenteron alaskense*, a synonym of *Le. kessleri*?

Because no character was found to distinguish the adults of *Le. kessleri* from those of *Le. alaskense*, we provisionally consider the latter to be a junior synonym of the former despite the fact that they occur on separate, but adjoining continents; *Le. kessleri* in Eurasia and *Le. alaskense* in North America. Further study is required to test this hypothesis. Interestingly, Berg (1948) had suggested that what he called *La. j. kessleri* was probably also present in Alaska and Poltorykhina (1974) treated the Alaskan *La. japonica* of Heard (1966) as *La. kessleri*. Additionally, in the original description of *Le. alaskense*, Vladykov and Kott (1978a) stated that they did not compare it to *Le. kessleri* because they lacked sufficient material for comparison. Holčík (1986c) treated *Le. alaskense* as a landlocked form of *Le. japonicum* (= *Le. camtschaticum*), but we reject this on the basis that the former is nonparasitic and the latter parasitic.

### *Lethenteron kessleri* distinct from *Le. reissneri*

A comparison of the original descriptions and extant type material of these two nominal taxa (this study; Renaud and Naseka 2015) did not reveal any significant differences other than total length (160–210 mm in *P. kessleri* versus 120–140 mm in *P. reissneri*), and this we believe is due to the incomplete descriptions and the poor condition of the specimens, in particular *Le. reissneri*, preventing a full comparison. However, Renaud and Naseka (2015) reported that the adult specimen identified by Berg (1931) and accepted by them as *Le. reissneri* (ZIN 14457) from the Shangshi River, Amur River drainage, does not have a blotch at the apex of its second dorsal fin and has a transverse lingual lamina with 2u–I–2u while the original description of *Petromyzon kessleri* Anikin, 1905 stated that the apex of the second dorsal fin is ash gray (i.e., blotched) and the transverse lingual lamina has a large median tooth flanked by eight or nine smaller teeth (i.e., 8u–I–8u or 9u–I–9u and combinations thereof). In two of the seven syntypes of *P. kessleri* for which we could determine the character state of the transverse lingual lamina (Table 2) these were 5u–I–7u and 7u–I–7u. Therefore, the pigmentation of the second dorsal fin and the transverse lingual lamina dentition are diagnostic characters for these two taxa. The suggestion by Yamazaki et al. (2006) that *Le. kessleri* is a synonym of *Le. reissneri* on the basis of similarities in their larval trunk myomere counts and identical electrophoretic profiles is thus rejected. Sato (1951) was the first to suggest that *Le. kessleri* (appeared as *La. j. kessleri*) was present on Hokkaido Island on the basis of 26 spawning adults 144–193 mm TL collected in the Shibechari River (now known as Shizunai River). However, we have re-identified this material as *Le. reissneri* because according to the description by Sato (1951) the specimens do not have a dark blotch on their second dorsal fin, but do have a pigmented caudal fin (++, +++; according to his fig. 1), an infraoral lamina bearing five to seven teeth, the lateralmost being bicuspid, usually three bicuspid endolaterals on each side, the third one rarely unicuspid on one or both sides, 17–23 teeth in the first posterial row, and 66–74 trunk myomeres. Additionally, material from the Shizunai River have an identical electrophoretic profile to *Le. reissneri* from the type locality of Onon River, Siberia (Yamazaki et al. 2006).

### *Lethenteron reissneri* present in the Angara River drainage, Russia

In their redescription of the species, Renaud and Naseka (2015) restricted the distribution of *Le. reissneri* to the Shilka and Songhua river systems within the Amur River drainage, until a more geographically comprehensive study is undertaken. Loshakova and Knizhin (2015) reported the presence of a nonparasitic lamprey in the geographically proximate Angara River drainage, Yenisei River system, Russia; specifically, in the Chuksha River. Their reported adult TL of 135–182 mm, caudal fin strongly pigmented [i.e., +++, based on figs 3, 4 in Loshakova and Knizhin (2015)], trunk myomeres 66–77, supraoral lamina with two unicuspid teeth, three bicuspid endolaterals on either side, infraoral lamina with usually four, less frequently five unicuspid teeth and one bicuspid lateralmost tooth on either side, 18–26 posterials, spawning at the end of May middle of June, absolute fecundity of 1,042–3,166 eggs with diameter 0.79–0.87 mm, all point to the identity being *Le. reissneri* and the reported absence of a dark blotch on the second dorsal fin distinguishes it from *Le. kessleri* (see above ‘*Lethenteron kessleri* distinct from *Le. reissneri*’, page 35). Indeed, Karasev (1987) reported an almost identical adult TL (i.e., 137–182 mm) for lamprey, identified as *Le. reissneri* by Renaud and Naseka (2015), from the upper Amur basin (Ingoda [type locality of *Le. reissneri*] and Shilka rivers), as well as a fecundity in females from the Ingoda River of 1,720–3,360 eggs with diameter 0.68–0.84 mm. Both syntypes of *Le. reissneri* examined by Renaud and Naseka (2015) also had a supraoral lamina with two unicuspid teeth and the only one in which endolaterals and trunk myomeres could be determined had three bicuspid endolaterals and 70 trunk myomeres. Although the only syntype in which the infraoral lamina could be studied had only one lateralmost bicuspid and five unicuspid teeth, another individual from the Shangshi River (Songhua River drainage, Amur River system), and identified by Berg (1931) as *Le. reissneri*, had two lateralmost bicuspid and four internal unicuspid teeth, as well as 24 posterials, a strongly pigmented (i.e., +++) caudal fin and no blotch on its second dorsal fin (see Renaud and Naseka 2015). Finally, Dybowski (1869) stated that *Le. reissneri* spawns in June. Unfortunately, the number of cusps on the transverse lingual lamina, another diagnostic character distinguishing *Le. reissneri* from *Le. kessleri*, was not recorded by Loshakova and Knizhin (2015). According to Enikeev (2018) a Transbaikalian paleolake existed in the Late Pleistocene connecting the Angara River to the upper Amur including the Onon and Ingoda rivers, the type locality of *Le. reissneri*, and we believe this could explain the present-day occurrence of this lamprey species in the Angara and Amur River drainages.

### *Lethenteron mitsukurii* distinct from *Le. kessleri* and *Le. reissneri*

*Lampetra mitsukurii* is placed by us in the genus *Lethenteron* because it possesses a row of posterials according to the original description (Hatta 1901) and the examination of two syntypes (Table 8). *Lethenteron mitsukurii* is distinct from *Le. kessleri* based on the absence of bicuspid lateralmost teeth on the infraoral lamina (Hatta 1901; Table 8) and the absence of pigmentation on its second dorsal and caudal fins (Table 9). The original description of *Le. kessleri* states that the lateralmost teeth on the infraoral lamina are bicuspid (Anikin 1905) and this was confirmed in five syntypes for which the character could be determined (Table 2). While examination of the pigmentary characters was uninformative in the syntypes of *Le. kessleri* (Table 3), the original description states that the apex of the second dorsal fin is ash gray (Anikin 1905) and an adult identified by Berg (1931) as *La. j. kessleri* from the Ob’ River (ZIN 6310, Table 6) has a heavily pigmented caudal fin (+++). *Lethenteron mitsukurii* is distinct from *Le. reissneri* based on syntypes of the latter possessing a bicuspid tooth on one or both sides of the infraoral lamina (Dybowski 1869; Renaud and Naseka 2015) and a heavily pigmented caudal fin (+++) in the adult from the Shangshi River, Amur River system (ZIN 14457) identified by Berg (1931) and accepted by Renaud and Naseka (2015) as *Le. reissneri*.

### Identity of the lamprey from Siberia described in Hatta (1901)

Hatta (1901) identified as *La. mitsukurii* a recently metamorphosed 150 mm TL female from Pervaya Rechka Brook near Vladivostok, Russia. Contrary to what is stated in Hatta (1901) this brook is not a tributary to the Amur River, but empties directly into the Sea of Japan. He notes that the first and second dorsal fins are separated by a deep notch; the supraoral lamina has two well separated blunt cusps; the infraoral lamina has six blunt cusps; three bicuspid endolaterals occur on each side; other labial teeth are simple and form a circle immediately inside the fringe of labial tentacles (= oral fimbriae). The latter character refers to the row of marginals. There is no mention of a row of posterials. Renaud and Naseka (2015) identified as *Le. camtschaticum* a 161.5 mm TL adult, previously identified by Berg (1931) as *La. reissneri*, from the Sedanka River emptying directly into the Sea of Japan near Vladivostok. Although the six unicuspid teeth on the infraoral lamina point to *Le. mitsukurii*, the absence of information on other key diagnostic characters (i.e., posterial row, second dorsal fin and caudal fin pigmentation) prevent us from confidently identifying as such the specimen from Siberia in Hatta (1901).

### *Lethenteron matsubarai*, a synonym of *Le. mitsukurii*

In our opinion *Le. matsubarai* is a junior synonym of *Le. mitsukurii* because both possess only unicuspid teeth on the infraoral lamina (Table 8) and unpigmented second dorsal and caudal fins (Table 9), three diagnostic characters that distinguish them from *Le. camtschaticum*, in which the infraoral lamina possesses at least one bicuspid tooth, the second dorsal fin has a blotch at its apex, and the caudal fin is strongly pigmented (Tables 2, 3). Likewise, the infraoral lamina of both *Le. reissneri* and *Le. kessleri* possess at least one bicuspid tooth (Renaud and Naseka 2015; Table 2). Unfortunately, the poor condition of the type material of *Le. reissneri* (see Renaud and Naseka 2015) and *Le. kessleri* (Table 3) did not permit an evaluation of the pigmentation of their second dorsal and caudal fins. However, Renaud and Naseka (2015) determined the second dorsal fin to be unpigmented (i.e., no blotch) and the caudal fin to be strongly pigmented in an adult of *Le. reissneri* from the Shangshi River (Amur River system), People’s Republic of China and Anikin (1905) described *Le. kessleri* as having the apex of the second dorsal and caudal fins pigmented, thus further distinguishing both species from *Le. mitsukurii*. Therefore, we reject the synonymy of *Le. matsubarai* with *Le. kessleri* proposed by Iwata et al. (1985), which was based on material presumed as belonging to the latter species that came from Hokkaido Island following Sato (1951) instead of examining type material or using the original description. The material identified as *Le. kessleri* by Sato (1951) has been re-identified by us as *Le. reissneri* (see above ‘*Lethenteron kessleri* distinct from *Le. reissneri*’, page 35). The material identified as *Le. kessleri* by Iwata et al. (1985) we believe to be a mixture of *Le. reissneri* and *Le. mitsukurii* because the authors report it to possess 65–73 trunk myomeres, an unpigmented second dorsal fin and a strongly pigmented caudal fin; a few individuals however having an unpigmented caudal fin. Those individuals with a strongly pigmented caudal fin correspond to *Le. reissneri*, while those with an unpigmented caudal correspond to *Le. mitsukurii*.

### Taxonomic identity of *Lampetra mitsukurii minor* and *La. m. major*

**Figure 5.**
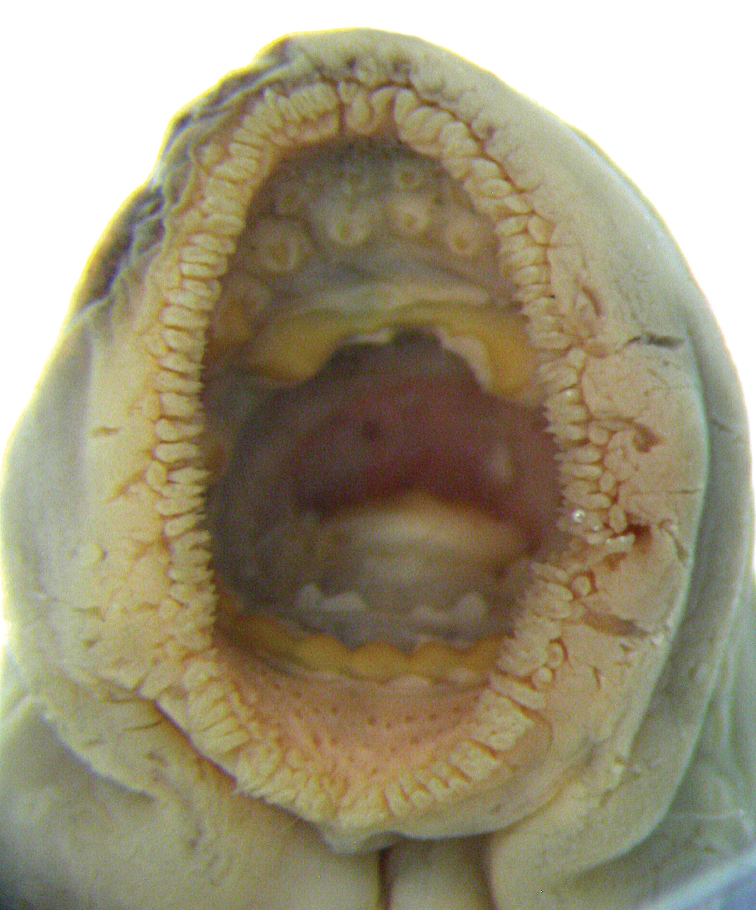
Oral disc of *Lethenteron reissneri*, ZIN 25204, 146 mm TL with two rows of posterials from Tym’ River, Sakhalin Island, Russia.

Hatta (1911) described two forms of *Lampetra mitsukurii*; one small (80–165 mm TL) with a widespread Japanese distribution he called *minor* and one large (350–410 mm TL) restricted to Sapporo, Hokkaido Island, he called *major*. The two forms occurred sympatrically on spawning grounds at Sapporo. Both were distinct from *La. japonica* (= *Le. camtschaticum*) based on characteristics related to sexual maturity (i.e., well-developed urogenital papilla in males and anal fin-like fold in females in the former versus undeveloped structures in both sexes in the latter, blunt cusps on the supraoral and infraoral laminae and thread-like intestines in the former versus sharp cusps on these laminae and relatively thick intestines in the latter). The subspecies minor is a junior synonym of *Le. mitsukurii* (Hatta, 1901) based on their very similar total lengths (80–165 versus 80–156 mm, respectively) and their identical geographic distributions (i.e., Hokkaido, Honshu, Shikoku, and Kyushu islands). The subspecies major is distinct from *La. mitsukurii* because figs 5 and 6 in Hatta (1911) showing close-ups of the cloacal regions of male and female *La. m. major*, respectively, possess a dark blotch at the apex of the second dorsal fin similarly to fig. 9 showing a close-up of the cloacal region of *La. japonica* (= *Le. camtschaticum*), whereas our study of a *La. mitsukurii* syntype revealed that its second dorsal fin is unpigmented (Table 9). Thus, even though Hatta (1911) states that the forms *minor* and *major* were often found attached to each other while spawning, they belong to distinct species. We therefore agree with Creaser and Hubbs (1922) and Berg (1931) that *La. m. major* is a synonym of what they respectively called Entosphenus (Le.) japonicus and *La. j. japonica* (= *Le. camtschaticum*). We disagree with Berg (1931) that *La. m. minor* is a synonym of *Le. reissneri* (see above ‘*Lethenteron mitsukurii* distinct from *Le. kessleri* and *Le. reissneri*’, page 37). Ioganzen (1935c) reported on adults of nine *La. m. minor* (130.7–162.3 mm TL) and two *La. m. major* (334.0–366.0 mm TL) identified by Prof. S. Hatta and collected sympatrically in Sapporo in April 1911. These were sent to Tomsk State University where they were studied in 1914 by the author’s father, Prof. G. E. Ioganzen. Unfortunately, in 1933 Ioganzen (1935c) could only find the *La. m. minor* and AMN could find neither when he visited TGU in 2011. According to Ioganzen (1935c) the supraoral lamina in both forms consisted invariably of two teeth while the infraoral lamina in *La. m. minor* possessed either 6(2), 7(4) or 9(1) unicuspid teeth or the formulae 5u1b(1) or 2b3u1b(1). Additionally, one count of six unicuspid teeth and three counts of seven unicuspid teeth are followed by unexplained exclamation marks and one count of seven unicuspid teeth is followed by an unexplained question mark. Both *La. m. major* possessed six teeth. However, since these two individuals were not personally examined by Ioganzen (1935c), we do not know whether the counts given represent only unicuspid or a mixture of unicuspid and lateralmost bicuspid teeth. While Hatta (1911) did not give tooth counts in his descriptions of *La. m. minor* and *La. m. major*, the presence of one or two bicuspid teeth on the infraoral lamina in two of the nine *La. m. minor* specimens is perplexing because it is different from the original description of *La. mitsukurii* by Hatta (1901) and our study of two syntypes (Table 8). Because this material of *La. m. minor* and *La. m. major* is now lost and relied in part on second-hand information, we treat it with suspicion and stand by our interpretations of the taxonomic identities of *La. m. minor* and *La. m. major*.

### *Lethenteron mitsukurii* not the spawning phase of *Le. camtschaticum*

Sato (1951) hypothesized that *Le. mitsukurii* was *Le. camtschaticum* (appeared as *La. j. japonica*) in its spawning phase. However, type material of *Le. mitsukurii* has unpigmented second dorsal and caudal fins (Table 9) and that of *Le. camtschaticum* is pigmented for both those characters (Table 3), and thus, we reject this hypothesis.

### Sympatric parasitic and nonparasitic taxa

According to Berg (1931) and Ioganzen (1935a, 1935b) the parasitic *La. j. septentrionalis* (= *Le. camtschaticum*) is distributed throughout the Ob’ River drainage, where it occurs sympatrically with the nonparasitic *La. j. kessleri* (= *Le. kessleri*). Specifically, Berg (1931) stated that *La. j. septentrionalis* (ZIN 6308: 355 mm TL) was sympatric with *La. j. kessleri* (ZIN 6307, ZIN 6311: 180–224 mm TL [ZIN 6311 as measured by us is very shriveled and only 144 mm instead of 180 mm as measured by Berg]) in the Irtysh River at Omsk and based on literature in the Tom’ River near Tomsk (the type locality of *P. kessleri*). In regard to the Tom’ River, Berg (1931) placed the 389 mm TL adult reported by Ruzskiy (1920) under the synonymy of *La. j. septentrionalis* contrasting with the 160–210 mm TL type series of *P. kessleri* reported by Anikin (1905). Ioganzen (1935a, 1935b) also stated that these two taxa were sympatric in the Tom’ River near Tomsk based on two adults of *La. j. septentrionalis* 215–408 mm TL and 12 adult syntypes of *P. kessleri* 132–207 mm TL. Six of the latter syntypes (TGU 9 = TGU 3696 in Ioganzen 1935b, table 3) were 128–165 mm TL as measured by us. In their study of the fishes of the Ob’-Irtysh system, Pavlov and Mochek (2006) reported *Le. camtschaticum* (appeared as *Le. japonicum*) to be sympatric with *Le. kessleri* in the Upper Ob’ and Middle Irtysh regions, as well as in the Southern and Northern Ob’-Taz Guba estuaries. The reported presence, albeit rare, of the nonparasitic *Le. kessleri* in estuarine waters is unexpected and may perhaps be explained by extensive freshwater plumes. It appears that the lower Tobol River, Ob’ River drainage, also contains both *Le. camtschaticum* and *Le. kessleri*. In the lower Tobol River Karasev (2008) reported (as *Le. kessleri*) five lamprey adults measuring 200–407 mm TL and seven mature females, the latter collected in late April and May, with absolute fecundity 3,161–7,208 eggs. We believe that the adults measuring over 230 mm TL (see above ‘Adult total length attained by *Le. kessleri*’, page 29) refer to *Le. camtschaticum* while the adults with the reported fecundity < 10,000 eggs refer to *Le. kessleri*. Unfortunately, Karasev (2008) did not report the total lengths of the females with the recorded fecundities. Contrary to the contention by Zhuravlev and Lomakin (2017) that anadromous *Le. camtschaticum* is presumed to have become extirpated from the upper Ob’ River drainage since the mid-20^th^ century, it would appear that it is still present (Pavlov and Mochek 2006; Karasev 2008). Further west, *Le. camtschaticum* and *Le. kessleri* are sympatric in the Severnaya Dvina River drainage (Berg 1931; Altukhov et al. 1958; Kuderskiy and Mel’nikova 1970; Sotnikov and Solov’ev 2002). According to Berg (1948)*Le. camtschaticum* and *Le. kessleri* are sympatric in the Pechora River drainage based on a 349 mm TL adult of the former from Shapochnaya (= Shapkina) River (ZIN 20802: 334 mm TL as measured by us, Table 4) and a 118 mm TL spent female of the latter from the Pechora River (ZIN 23909: 110 mm TL as measured by us). *Lethenteron camtschaticum* and *Le. kessleri* are also sympatric in the Yenisei River based on our re-interpretation of *P. dentex* as a junior synonym of *Le. camtschaticum* (see above ‘Real or perceived distributional discontinuity between the populations of parasitic *Le. camtschaticum* in Russia?’, page 28) and one individual (ZIN 14441) of *Le. kessleri* (Tables 4–6). Nazarov (2012) also reports Siberian lamprey (= *Le. kessleri*) in the middle Yenisei (i.e., Chernaya and Beryozovka rivers) based on 152 adults 122–211 mm TL. *Lethenteron camtschaticum* and *Le. kessleri* are sympatric in the Utkholok River drainage, Kamchatka (see above ‘Identity of lampreys in the Utkholok River drainage, Kamchatka’, page 31) based on our re-interpretation of the data presented in Kucheryavyi et al. (2007a). *Lethenteron camtschaticum* and an unidentified nonparasitic species very similar to *Le. mitsukurii* are sympatric in the Kol’ River drainage, Kamchatka (see above ‘Identity of lampreys in the Kol’ River drainage, Kamchatka’, page 33). *Lethenteron camtschaticum* and an unidentified nonparasitic species are also sympatric in Lake Azabach’e basin, Kamchatka (see above ‘Identity of lampreys in the Lake Azabach’e basin, Kamchatka’, page 34). On Sakhalin Island, *Le. camtschaticum* is sympatric with nonparasitic *Le. reissneri* in the Tym’ River (Gritsenko 1968, 2002; Dyldin and Orlov 2016; this study – see below ‘Taxonomic identity of nonparasitic lampreys in Japan and Sakhalin Island, Russia with low trunk myomere counts’, page 42). While Gritsenko (2002) recorded the presence of another nonparasitic species in the Tym’ River, *Le. kessleri*, the identity of the species was not clearly established because he used the number of trunk myomeres to distinguish it from *Le. reissneri* and the number recorded (i.e., 64–74) could apply to either species as the type material of *Le. kessleri* possessed 70–74 (this study) and that of *Le. reissneri* possessed 70 (Renaud and Naseka 2015). Interestingly, out of several hundred specimens of brook lamprey from the Tym’ River examined by Gritsenko (2002), three possessed two rows of posterials, like the specimen of *Le. reissneri* mentioned above that we identified from that river. Unfortunately, the key diagnostic characters separating the two species (i.e., the pigmentation of the second dorsal fin and the transverse lingual lamina dentition; see above ‘*Lethenteron kessleri* distinct from *Le. reissneri*’, page 35) were not recorded. In the People’s Republic of China, *Le. camtschaticum* (as *La. japonica*) is sympatric with nonparasitic *Le. reissneri* in the Mudan River (not Mutantiang River), tributary to Sungari (Songhua) River (Hensel 1963). Li and Liu (2011) confirm the presence of *Le. camtschaticum* (as *La. japonica* or *Le. japonicum* used interchangeably) in the Songhua River system based on prespawning adults 385–470 mm TL. In Japan, *Le. camtschaticum* is sympatric with nonparasitic *Le. mitsukurii* in Sapporo, Hokkaido Island (see above ‘Taxonomic identity of *Lampetra mitsukurii minor* and *La. m. major*’, page 38) and Shokotsu River, Hokkaido Island (Vladykov and Kott 1978b in which *Le. matsubarai* is a synonym of *Le. mitsukurii*). Additionally, Yamazaki et al. (1998) reported the presence of mature dwarf male and female *Le. camtschaticum* (as *Le. japonicum*), which they suggested were nonparasitic, together with anadromous *Le. camtschaticum* (also as *Le. japonicum*) in the Ohno River, Hokkaido Island, Japan. However, neither photograph of a male and a female dwarf individual in their figure 1 exhibit a dark blotch on the second dorsal fin, and therefore, these would not be *Le. camtschaticum*, but rather belong to an unidentified nonparasitic species. Later, Yamazaki and Goto (2016) provided another photograph of a mature nonparasitic lamprey, measuring 140–160 mm TL, from the Ohno River (their photo 2–1B) showing a dark blotch on the second dorsal fin and a heavily pigmented caudal fin, which in our opinion could be *Le. kessleri*, but this needs confirmation. Yamazaki et al. (2011) and Yamazaki and Goto (2016) referred to the nonparasitic lamprey from the Ohno River as a population of fluvial *Le. camtschaticum*. In Lake Sopochnoe basin, Iturup Island, Kuril Archipelago, Russia, *Le. camtschaticum* is sympatric with *Le. kessleri* (Sidorov and Pichugin 2005). However, these authors used the identification criteria of Iwata et al. (1985) and we have argued above (see ‘*Lethenteron matsubarai*, a synonym of *Le. mitsukurii*’, page 38) that the specimens identified as *Le. kessleri* by the latter represented a mixture of *Le. reissneri* and *Le. mitsukurii.* Unfortunately, Sidorov and Pichugin (2005) did not comment on the pigmentation of the caudal fin, and therefore, we cannot make a definitive identification between these two species. In Alaska, *Le. camtschaticum* (as *Le. japonicum* in Vladykov and Kott 1978a) is sympatric with nonparasitic *Le. alaskense* (= *Le. kessleri*?; see key below, page 53) in the Naknek River system (Vladykov and Kott 1978a), as well as the Chatanika and Chena rivers, Yukon River drainage (Vladykov and Kott 1978a; Sutton 2017).

### Taxonomic identity of nonparasitic lampreys in Japan and Sakhalin Island, Russia with low trunk myomere counts

The nonparasitic *Le. reissneri* has long been reported from Japan (Berg 1931, 1948; Mori 1936; Okada 1960; Hubbs and Potter 1971; Vladykov and Kott 1978a, 1978b; Sato 1984). However, in the redescription of this species Renaud and Naseka (2015) restricted its distribution to mainland Asia (Russia, Mongolia, People’s Republic of China) pending a more comprehensive study across a wider geographic range, because of a marked difference in the number of trunk myomeres between *Le. reissneri* from mainland Asia versus nonparasitic lamprey in Japan. Indeed, the number of trunk myomeres in adult *Le. reissneri* (including a syntype) from mainland Asia is 70–72 (Renaud and Naseka 2015) versus 57–63 in adults referred to this species from Japan and Sakhalin Island (Vladykov and Kott 1978a). There are two available names for nonparasitic species described from Japan, *Le. mitsukurii* and its junior synonym *Le. matsubarai*. Their adults possess 66–70 trunk myomeres (Table 8; Vladykov and Kott 1978b), higher counts than the 57–63 recorded for Japanese “*Le. reissneri*” by Vladykov and Kott (1978a). Interestingly, the basis for the first report of *Le. reissneri* in Japan was the synonymy by Berg (1931) of *Le. mitsukurii* with *Le. reissneri* due to his re-identification of Japanese adult lamprey 86–147 mm TL (erroneously reported as 92–145 mm TL) with 56–67 trunk myomeres identified as *Le. mitsukurii* (appeared as *Entosphenus mitsukurii*) by Jordan and Hubbs (1925). Hubbs and Potter (1971) had remarked that unidentified nonparasitic lampreys from Hokkaido (Japan) and Siberia (Russia) possessed ca. 70 or more trunk myomeres, contrasting with the lower counts of Jordan and Hubbs (1925). It is important to note that Berg (1931) did not examine type material of *Le. mitsukurii* or *Le. reissneri* in reaching his conclusion. Yamazaki and Goto (1996) discovered fixed alternate allelic differences at eleven loci (AAT–1, G6PDH, GPI–2, IDHP–1, IDHP–2, IDHP–3, IDHP–4, MDH–3, MDH–4, MEP–1, PGM) among what they assumed were Japanese populations of *Le. reissneri* collected from 27 rivers across Hokkaido and Honshu islands, which they identified as northern (N) and southern (S) groups. Furthermore, they detected no evidence of hybridization between three sympatric river populations on Honshu Island (i.e., Uono, Shou-gawa, and Shoushu rivers) implying that the northern and southern groups were reproductively isolated. Yamazaki et al. (1999) extended the range of the S-group to include Shikoku and Kyushu islands, Japan, and the Korean Peninsula. They also added five other rivers on Honshu where the two forms (N and S) were sympatric (Ushiwatari, Takifuchi, Shouzenji, Makino, and Shourai) and Yamazaki et al. (2001a) added a further one (Gakko). Despite an overlap in the spawning season and size at spawning of the two forms in the Ushiwatari River, tributary to the Gakko River, Yamazaki and Goto (2000b) found only males and females of a given form on their own nests and no evidence of gene exchange between the two forms, which they suggested may be due to an unidentified premating isolating mechanism ensuring that they remain distinct. Additionally, Yamazaki et al. (2003) found differences in the mtDNA COI sequences (1095 bp) between the N and S forms. Interestingly, the mean percentage sequence differences between them (9.1%) was greater than between either of them and *La. fluviatilis* (7.0–8.8%). However, Yamazaki and Goto (1997) could find no diagnostic morphometric, countable (trunk myomere and dentition) or pigmentation (caudal fin) characters to distinguish adults of the two. Yamazaki and Goto (1997) state that the caudal fin of the northern and southern groups is translucent or slightly pigmented. This is highly significant because the caudal fin pigmentation of *Le. reissneri* is heavily pigmented (+++) according to Renaud and Naseka (2015), while it is unpigmented (-) in *Le. mitsukurii* (Table 9). Additionally, the range in adult total length reported by Yamazaki and Goto (1997) for the northern and southern groups combined (87.9–169.1 mm) is very similar to that (80–156 mm) reported in the original description of *Le. mitsukurii*. According to Yamazaki et al. (2006) the two undescribed *Lethenteron* spp. from Japan are distinguished from *Le. reissneri* of the upper Amur River system (from the type locality) on the basis of the number of larval trunk myomeres: 65–73 (upper Amur *Le. reissneri*), 51–66 (*Lethenteron* sp. N), and 49–62 (*Lethenteron* sp. S). Yamazaki et al. (2006) further added that *Le. reissneri* from the type locality (Onon and Ingoda rivers) and *Le. * sp. S from the Naktong (= Nakdong) River, South Korea are fixed for alternate alleles at eight loci (AAT–1, IDHP–1, IDHP–2, IDHP–3, IDHP–4, MDH–3, MDH–4, PGM). Remarkably, based on a strict consensus tree generated from maximum parsimony of a partial sequence of the mtDNA COI gene (1009 bp), *Le. * sp. N is sister to a *Lethenteron* clade (100% bootstrap support) that includes *Le. japonicum* (= *Le. camtschaticum*), *Le. reissneri*, and *Le. kessleri*, while *Le. * sp. S is sister to an *Entosphenus*–*Lampetra*–*Lethenteron* clade (99% bootstrap support) that also includes *Le. * sp. N (Yamazaki et al. 2006). Yamazaki and Goto (1998) conducted an electrophoretic analysis of *Le. japonicum* (= *Le. camtschaticum*) and *Le. kessleri* collected from Hokkaido and Honshu islands, Japan, and the Far Eastern region of Russia. These were identified based on morphological features reported by Iwata et al. (1985). Although Yamazaki and Goto (1998) found that the two species were fixed for alternate alleles at one locus (MDH–3) at the three Hokkaido Island localities (Sarufutsu, Hororo, and Mena rivers) where they were sympatric, the identity of *Le. kessleri* is in question because it did not possess a pigmented second dorsal fin as reported in the original description and none of its samples came from the type locality. Yamazaki et al. (2006) found that lamprey material from the type locality of *Le. reissneri* (Onon and Ingoda rivers, Russia) and from the Ob’ River drainage, the same river drainage as for the type locality of *Le. kessleri* (Tom’ and Kirgizka rivers, Russia), although very distantly removed from the latter (Irtysh and Uba rivers, Kazakhstan), shared the same MDH–3 allele. However, we have shown above that two morphological characters (transverse lingual lamina counts and pigmentation of the second dorsal fin) distinguish those two species. At this point, only two nonparasitic species are confirmed to occur in Japan: *Le. reissneri* (Shizunai River, Hokkaido Island, this study) and *Le. mitsukurii*. As adults of both *Le. mitsukurii* and *Le. reissneri* have high trunk myomere counts (66–72: this study; Renaud and Naseka 2015), an in-depth study of large samples of nonparasitic lamprey adults from Japan and Sakhalin Island is needed to determine whether the lower trunk myomere (< 66) individuals from these areas represent one or more undescribed species, or *Le. mitsukurii*, or *Le. reissneri*, or a mixture of these three alternatives. Yamazaki and Goto (2000a) made the suggestion that perhaps *Lethenteron* form N or *Le. * form S could in fact be *Le. mitsukurii*. Recently, using data extracted from two theses, Dyldin et al. (2019b) could not determine the specific identity of adult *Lethenteron* lampreys from four Sakhalin rivers (Bol’shoy Garomai, Nitui, Novikovka, Pugachovka) measuring 116–220 mm TL and with 65–75 trunk myomeres. In this regard we have identified as *Le. reissneri* a 146 mm TL (158 mm originally) adult with 68 trunk myomeres from Tym’ River, Sakhalin Island, Russia (ZIN 25204) that was treated as *Lethenteron* sp. in Tuniyev et al. (2016). Although placed under the *La. reissneri* species account in Berg (1948), he added (p. 43, fig. 30) that it was possibly a new species because it possessed multiple posterial rows. Two posterial rows have been reported by Renaud and Naseka (2015) in a specimen of *La. reissneri* from the Shangshi River, People’s Republic of China (ZIN 14457). We diagnosed the specimen from Sakhalin (Fig. 5) as *Le. reissneri* because it possesses two rows of posterials, the first one with 20 unicuspid teeth, the endolateral formula is 2–2–2, the infraoral lamina is 1b5u1b, the second dorsal fin is unpigmented and the caudal fin heavily pigmented (+++). According to Nikoforov et al. (1994), the paleo-Amur was hypothesized to be connected to the eastern littoral of Sakhalin Island in the geological past, and we believe this may explain the presence of *Le. reissneri* in the Tym’ River in present times. One of the syntypes of *La. mitsukurii* (Table 8) also possessed two rows of posterials indicating that this condition is not restricted to *Le. reissneri*.

### Synonymy of *Lethenteron camtschaticum* (Tilesius, 1811), the Arctic lamprey

Synonyms, new combinations, and misidentifications are included.

*Petromyzon marinus Camtschaticus* Tilesius, 1811: 240–247, pl. IX, figs I, II [original description, marine waters of St. Peter and Paul Camtschatici (= Petropavlovsk-Kamchatsky) harbor, Russia].

*PetromyzonFluvialis* (non Linnaeus, 1758) – Richardson 1823: 705 [misspelling of *P. fluviatilis* Linnaeus, 1758; common name: Lesser lamprey, Great Slave Lake, Northwest Territories, Canada, attached to an Inconnu, *salmo Mackenzii* (= *Stenodus leucichthys*)]; Richardson 1836: 294 [common name: River lamprey, refers to the individual in the previous reference].

*Petromyzon borealis* Girard, 1858: 377 [available by indication to *Petromyzon fluviatilis* Richardson, 1836].

*PetromyzonJaponicus* von Martens, 1868: 3–5, pl. I, fig. 2 [original description, Tokyo (appeared as Jeddo) and Yokohama, Honshu Island, Japan].

*Petromyzon Kameraticus* – Dybowski, 1869: 948 [misspelling of *P. camtschaticus* Tilesius, 1811; treated as a distinct species, not a subspecies of *P. marinus* as Tilesius (1811) intended, ascends to Stretensk on the Shilka River, a tributary of the Amur River, Russia].

*Petromyzon fluviatilis* (non Linnaeus, 1758) – Günther 1870: 504 [presence of a transverse series of small teeth behind the mandibular tooth (= row of posterials) in *Petromyzon japonicus* von Martens, 1868 from Japan judged insufficient by Günther (1870) to distinguish it from *P. fluviatilis*.]

*Petromyzon Ernstii* Dybowski, 1872: 220 [original description, mouth of the Amur River, Russia].

*Petromyzon Kameralicus* – Dybowski 1872b: 221 [misspelling of *P. camtschaticus* Tilesius, 1811].

*Petromyzon kamtschaticus* – Dybowski 1872b: 221 [misspelling of *P. camtschaticus* Tilesius, 1811].

*Ammocoetes aureus* Bean, 1881: 159 [original description, Yukon River at Anvik (63°N, 160°W), Alaska, U.S.A.].

Petromyzon (Ammocoetes) fluviatilis var. (non Linnaeus, 1758) – Smitt 1895: 1190–1191, fig. 353 [Archangel, Russia, 315 mm TL male with middle pair of the lateral (= endolateral) teeth bicuspid and a curved but irregular row of teeth in the posterior part of the disc (= row of posterials)].

*Lampetra aurea* – Jordan and Evermann, 1896: 13 [comb. nov., Yukon River]; Jordan and Gilbert, 1899: 434 [Yukon River]; Schmidt 1904: 336 [northern Bering Sea]; Evermann and Goldsborough, 1907: 222, 227 [common name: Lamprey eel, Bering Sea basin].

*Entosphenus camtschaticus* – Jordan and Gilbert, 1899: 434 [comb. nov., allocation to *Entosphenus* is proposed as probable, but not certain, Kamchatka].

*Lampetra japonica* – Hatta 1901: 22–24 [comb. nov., Honshu and Hokkaido islands, Sea of Japan basin, Japan]; Jordan and Snyder 1901b: 733 [Japanese common name: Yatsumeunagi, which translates to eight-eyed eel, 470 mm TL, Shinano River, Honshu Island, Japan]; Soldatov and Lindberg 1930: 2–3 [common name: Japanese lamprey, adults up to 350 mm TL, but fig. 1 indicates 452 mm, Tumen River, Maikhe River mouth, Ussuri Bay, Amur estuary, Vorovskaya River (Kamchatka), Russia]; Mori 1936: 3, 6, 10, 15 [Maritime, Karafuto (= Sakhalin), Hokkaido, Amur zoogeographical districts, Japan, Chosen (= Korea)]; Berg 1948: 25, 29–34 [Arctic and Pacific Ocean basins]; Ivanova-Berg and Manteyfel’ 1949: 18 [prespawning adults feed on blood and muscle of *Coregonus nasus* in Gulf of Ob’ and Tazovskaya Bay and *Clupea pallasii* in Severnaya Dvina mouth and Pechorskaya Bay]; Birman 1950: 158–159 [common name: Pacific lamprey, lamprey marks on *Oncorhynchus gorbuscha* in the Amur River estuary and mouth of the Tumnin River, Russia, and less frequently on *O. keta*, in the Amur River estuary]; Walters 1955: 267, 272 [common name: Arctic lamprey, 355 mm TL adult, Point Barrow, Alaska, U.S.A.]; Morozova 1956: 149 [common name: Pacific lamprey, spawning run on Amur River near Elabuga and Malmyzh, Russia, 3 Dec. 1948 – 8 Jan. 1949, 6–30 Dec. 1949]; Nikol’sky 1956: 588–590 [common name: Pacific lamprey, 22 adults 147–293 mm TL, with a blue-gray dorsal aspect and silvery-white ventral aspect, except for the largest one in which the dorsal aspect is greenish and the ventral aspect yellowish, feeding on *Osmerus dentex* on 11 Aug. 1955 in marine waters two to three km off Ribnovsk, Sakhalin Island, Russia; their intestines contained *O. dentex* scales, muscle, intestine, gonad, and bones]; Heard 1966: 332, 334, 336, 338 [in part, common name: Arctic lamprey, presumed anadromous, parasitic on *Gasterosteus aculeatus*, *Prosopium coulterii*, *Oncorhynchus mykiss*, *O. nerka*, mature or spent adults 218–311 mm TL, Naknek River and Brooks River, Naknek River system, Alaska, U.S.A.]; Gritsenko 1968: 157 [lamprey marks on anadromous *Oncorhynchus gorbuscha*, *O. keta*, *O. kisutch*, *O. masou*, *Salvelinus alpinus* entering Tym’ River, Sakhalin Island, Russia]; McPhail and Lindsey 1970: 50–55 [in part, common name: Arctic lamprey, adults from Alaska ca. 90–411 mm TL some feeding on *Oncorhynchus tshawytscha*, *Platichthys stellatus*; according to map, Alaskan waters of the Bering Sea basin from the Alaska Peninsula northwards to St. Lawrence Island, U.S.A., Beaufort Sea basin from near Barrow, Alaska, U.S.A. to Anderson River, Northwest Territories, Canada, Yukon River and off Herschel Island (feeding on *Osmerus dentex*, *Stenodus leucichthys*), Yukon, Canada, Great Slave Lake basin northward to Mackenzie River estuary, eastward to Artillery Lake and southward to Slave River at Fort Smith, Northwest Territories, Canada]; Savvaitova and Maksimov 1978: 556 [230–320 mm TL spawning individuals 17–21 June 1972 in the Levyy Kolkalvayam River, tributary to the Utkholok River, western Kamchatka, Russia]; Sato 1984: 2 [Japanese common name: Kawa-yatsume, parts of second dorsal and caudal fins blackish in adults, Japan]; Novomodnyy and Belyaev 2002: 81 [young lamprey adults ≤ 210 mm in length feed on *Oncorhynchus gorbuscha* and *O. keta* smolts ≤ 85 mm in length and *O. masou* ≥ 120 mm in length in the Amur River estuary and Sakhalin Bay, Russia]; Li et al. 2019: 1501–1502, 1505, 1507, 1509–1512 [Tumen and Amur rivers, People’s Republic of China, early development].

*Lampetra mitsukurii* (non Hatta, 1901) – Jordan and Snyder 1901b: 734 [in part, 305–356 mm TL, Ishikari River, Sapporo, Hokkaido Island, Japan].

*Entosphenes camtschaticus* – Schmidt 1904: 336 [misspelling of *Entosphenus*, brackish-water form, Bering Sea off Kamchatka].

*Petromyzon dentex* Anikin, 1905: 15–17 [original description, mouth of the Yenisei River, near Gol’chikha, Russia].

*Lampetra fluviatilis* (non Linnaeus, 1758) – Berg 1906: 177, 179 [in part, ZIN 7814].

**Figure 6.**
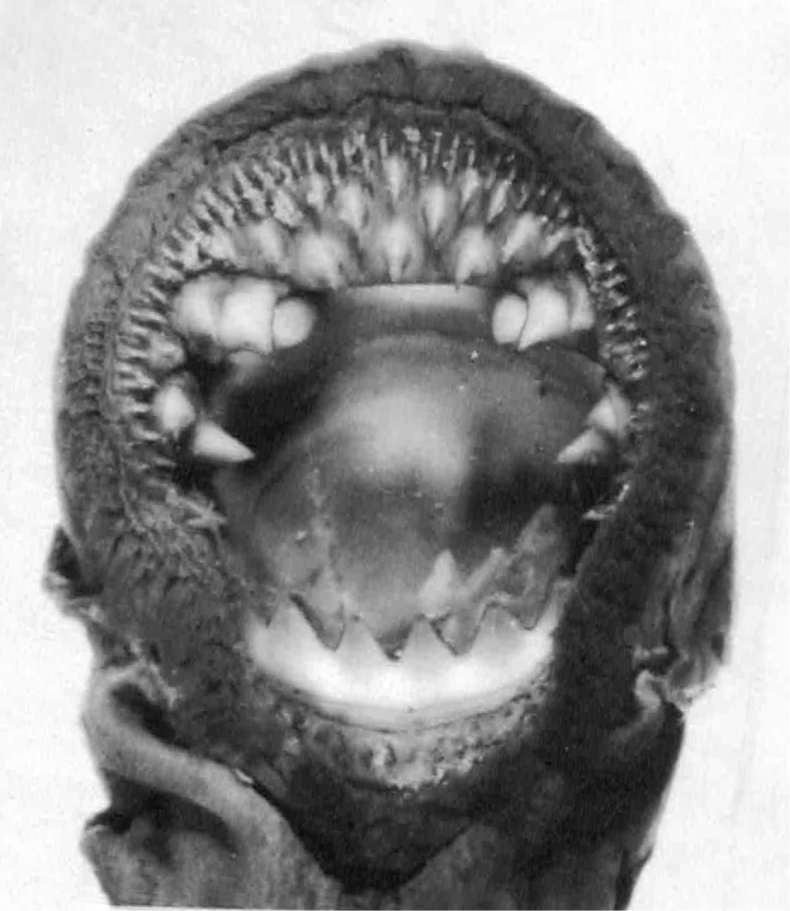
Oral disc of syntype of *Lampetra morii*, CMNFI 1986–757 (formerly part of ZIN 23145), 171 mm TL. Photograph by George Ben-Tchavtchavadze, University of Ottawa, pre–1982.

*Lampetra mitsukurii major* Hatta, 1911: 266–268, pl. IX, figs 1, 2, 5, 6 [original description, Sapporo, Hokkaido Island, Japan, spawning male and female].

*Entosphenus japonicus* – Regan 1911: 201–202 [comb. nov., Echigo Province (= Niigata Prefecture minus Sado Island), Tokyo, and Hokkaido Island, Japan; Archangelsk, Russia]; Jordan et al., 1913: 6 [Japanese common name: Kawayatsume, northern Japan]; Jordan Hubbs 1925: 98 [supraoral lamina with a cusp at each end and at most a minute cusp on the bridge; infraoral lamina with six to eight teeth, the lateralmost bicuspid and the internal ones unicuspid; reported locality is Karafuto, near Otaru, Hokkaido Island, Japan, but this makes no sense as Karafuto is the former name of the southern part of Sakhalin Island].

*Lampetra fluviatilis japonica* – Berg 1911: 33–34 [comb. nov., ZIN 6308, 7814, 8545, 12159, 14371]; Ruzskiy 1920: 30 [common name: Siberian-Japanese lamprey, 389 mm TL adult, Tom’ River near Tomsk, Russia]; Soldatov 1924: 16 [common name: River lamprey, 330 mm adult in a drift net at the freshwater/brackish water interface of the Pechora River mouth, Russia]; Knipovich 1926: 51 [common name: Siberian river lamprey].

Entosphenus (Lethenteron) japonicus – Creaser and Hubbs 1922: 3, 6–7, 11 [new subgenus based on *Lampetra wilderi* Gage in Jordan & Evermann, 1896; coasts and streams from Bering Sea west to the White Sea and south to the Sea of Japan].

*Lampetra borealis* – Jordan et al. 1930: 10 [comb. nov., common name: Arctic lamprey, streams of northern Alaska and Kamchatka].

Lampetra (Lampetra) japonica septentrionalisBerg 1931: 93, 100–102, pl. V, fig. 4 [original description and key, Onega River at Podporozh’e, White Sea basin, Russia].

Lampetra (Lampetra) japonica japonica – Berg 1931: 93, 98 [nominative subspecies based on *PetromyzonJaponicus* von Martens, 1868].

Lampetra (Lampetra) japonica kessleri (non Anikin, 1905) – Berg 1931: 102 [Anadyr Liman (= Estuary), Russia; ZIN 23154, 258 mm TL spent female collected in brackish water attached to *Onchorhynchus keta*; ZIN 23158, two adults 135–138 mm TL with very sharp teeth collected in brackish water and attached to *O. keta*]; Berg 1948: 35 [Anadyr Estuary, Russia: two uncatalogued specimens with sharp teeth and thick intestines; one (155 mm TL) attached to a sculpin (Cottidae) and the other (144 mm TL) to *O. keta*].

Lampetra (Lampetra) reissneri (non Dybowski, 1869) – Berg 1931: 104 [Samarga (ZIN 15078) and Sedanka (ZIN 15747) rivers, Russia].

*Lampetra fluviatilis septentrionalis* (non Berg, 1931) – Ioganzen 1935a: 369 [comb. nov.].

*Lampetra japonica japonica* – Taranetz 1937: 47 [common names: Pacific lamprey, Japanese lamprey]; Shmidt 1950: 16, 232, 236 [Okhotsk Sea basin: mouth of Amur River, Gulf of Sakhalin, northern part of the Sea of Okhotsk, western coast of Kamchatka, eastern and northern coasts of Sakhalin Island, Russia].

*Entosphenus japonicus septentrionalis* – Rawson 1951: 208, 221 [comb. nov., common name: Northern lamprey, Great Slave Lake in Fort Resolution area, near town of Hay River and Gros Cap, Northwest Territories, Canada; the latter two in *Lota lota* and *Stenodus leucichthys* stomachs, respectively].

*Entosphenus lamottei japonicus* – Wilimovsky 1954: 281 [comb. nov., Arctic

Alaska, Bering Sea, north and east Asia].

Lampetra (Lethenteron) japonica – Hubbs and Potter 1971: 51 [comb. nov., Varanger Fjord throughout Siberia, along the eastern Pacific coast, Japan, Alaska, and northern Canada].

*Lethenteron japonicum* – Nursall and Buchwald 1972: iv, 14, 18, 24 [comb. nov., common name: Arctic lamprey, adults 168 to > 300 mm TL; Great Slave Lake, Slave River, Hay River, Northwest Territories, Canada; host of *Catostomus catostomus*, *Coregonus artedi*, *C. clupeaformis*, *Lota lota*, *Salvelinus namaycush*, *Stenodus leucichthys* in fresh water]; Vladykov and Kott 1978a: 3, tables 9–10, 22 [Mackenzie River drainage, Northwest Territories, Canada; Beaufort Sea; Naknek River system, Nushagak and Yukon rivers, Alaska, U.S.A; Honshu and Hokkaido islands, Japan; Amur River, Russia]; Iwata et al. 1985: 185–186 [Saru, Mu, Ryukei rivers and Tofutsu Lake, Hokkaido Island, Japan]; Iwata and Hamada 1986: 17–20 [common name: Arctic lamprey, 197 mm TL adult male with fully pigmented caudal fin and blotch on second dorsal fin, Assabu River, Hokkaido Island, Japan; 153–228 mm TL young adults, Tofutsu Lake and Saru River, Hokkaido Island, Japan; 365–533 mm TL adult males, Assabu, Hime, Toshibetsu rivers, Hokkaido Island and Mabechi River, Honshu Island, Japan]; Yamazaki et al. 2001b: 1135 [Japan: Ishikari, Ohno, Saru, and Uzura rivers, Hokkaido Island, 215.2–478.0 mm TL adults; Mogami River, Honshu Island, 397.0–431.0 mm TL adults]; Martynov 2002: 145 [common name: Pacific lamprey, upstream migrants in the Vashka River, Mezen’ River drainage, Russia, mean TL 417 mm for males and 424 mm for females, one out of 109 adults had a third cusp on the bridge of its supraoral lamina and two out of 111 adults had unicuspid instead of bicuspid lateralmost teeth on the infraoral lamina]; Gritsenko 2002: 13–21 [340–570 mm TL adults, Tym’ River, and lamprey marks on anadromous *Oncorhynchus gorbuscha*, *O. keta*, *O. kisutch*, *O. masou*, *Salvelinus alpinus*, *S. leucomaenis* entering Tym’ River and Nyyskiy Bay, Sakhalin Island, Russia]; Reshetnikov 2002: 28 [intestinal diameter in adult 4–20 mm].

*Lethenteron japonica* – Nikoforov et al. (1994): 26 [common name: Arctic lamprey, southern Sakhalin Island, Russia].

Lampetra (Lethenteron) camtschatica – Mecklenburg et al. 2002: 62 [comb. nov., common name: Arctic lamprey, Alaska and elsewhere in the northern hemisphere, but not Caspian Sea as erroneously stated].

*Lethenteron camtschaticum* – Sidorov and Pichugin 2005: 402–405 [common name: Japanese lamprey, silvery downstream migrants to the sea, 151–201 mm TL, feeding on *Oncorhynchus gorbuscha*, *O. nerka*, *Salvelinus malma*, Lake Sopochnoe basin, Iturup Island, Kuril Archipelago, Russia, 3–10 Aug. 2001]; Gritsenko et al. 2006: 16–17 [common name: Pacific lamprey]; Kucheryavyi et al. 2007a: 41–44 [in part, Kolkavayam and Utkholok rivers, western Kamchatka, Russia, typically anadromous form, 174–350 mm mature individuals of both sexes combined]; Bugayev et al. 2007: 32–33 [common name: Pacific lamprey, 310–320 mm TL adults, Kamchatka River estuary, Russia]; Sutton 2017: 1198 [anadromous, adults, 304–427 mm TL, Chatanika and Chena rivers, Yukon River drainage, Alaska, U.S.A.]; Chereshnev 2008: 25–31 [common name: Pacific lamprey, 310 mm TL individual from the stomach of a burbot, *Lota lota*, in the middle reaches of the Anadyr River, Russia]; Shevlyakov and Parensky 2010: 396, 399 [common name: Kamchatka lamprey, lamprey wounds and scars 6–22 mm in diameter above the lateral line between the dorsal and adipose fins of *Oncorhynchus keta* in the lower Kamchatka River and Kamchatka Bay, Russia]; Novikov and Kharlamova 2018: 296, 298–301 [common name: Arctic lamprey, adults 230–480 mm TL, Barents Sea up to 76°N and White Sea at depths 10–131 m]; Siwicke and Seitz 2018: 111 [common name: Arctic lamprey, 28 feeding individuals collected using a epipelagic trawl in the eastern Bering Sea at depths < 100 m between mid-August and early October 2012]; Shink et al. 2019: 1993–1998 [common name: Arctic lamprey, feeding adults, 187–465 mm TL, on *Mallotus villosus*, *Clupea pallasii*, *Ammodytes hexapterus*, *Eleginus gracilis*, *Gadus chalcogrammus*, *Leptoclinus maculatus*, *Limanda aspera*, *Oncorhynchus gorbuscha*, *O. tshawytscha*, *Osmerus dentex*, Cottidae, Gasterosteidae, eastern Bering Sea, Alaska, U.S.A.].

*Lampetra camtschatica* – Sawatzky et al. 2007: 10, 12–13 [in part, common name: Arctic lamprey, Northwest Territories, Canada, including Horton River].

*Lethenteron reissneri* (non Dybowki, 1869) – Bugayev et al. 2007: 33–34 [two males, 185 mm TL, one from Lake Azabach’e and one from Lake Kurzin, both in the Kamchatka River drainage, Russia with half-digested fish flesh in their intestines].

### Synonymy of *Lethenteron mitsukurii* (Hatta, 1901), the Japanese brook lamprey

Synonyms, new combinations, and misidentifications are included.

*Lampetra mitsukurii* Hatta, 1901: 22–24 [original description, Hondo [= Honshu], Shikoku, Kyushu, and Hokkaido islands, Japan]

*Lampetra mitsukurii* – Jordan and Snyder 1901a: 126 [Japanese common name: Sunayatsume].

*Lampetra mitsukurii minor* Hatta, 1911: 263–266, 268, pl. IX, figs 3, 4, 7, 8 [trinomial based on *Lampetra mitsukurii* Hatta, 1901].

*Lampetra planeri* (non Bloch, 1784) – Regan 1911: 203 [misidentification based on synonymy with *Lampetra mitsukurii* Hatta, 1901]; Jordan et al. 1913: 6 [Japan]; Creaser and Hubbs 1922: 13 [Japan].

*Entosphenus appendix* (non DeKay, 1842) – Creaser and Hubbs 1922: 7, 12 [in part, eastern Asia, including Japan].

*Entosphenus mitsukurii* – Hubbs 1925: fig. 16, 589 [comb. nov., a degenerate, dwarf, brook lamprey derived from anadromous, parasitic *Entosphenus japonicus*]; Jordan and Hubbs 1925: 98–99 [in part, mature male, 147 mm TL, 67 trunk myomeres, mottled coloration and mature female, 142 mm TL, 63 trunk myomeres, plain coloration, Sapporo, Hokkaido Island, Japan].

Lampetra (Lethenteron) mitsukurii – Hubbs and Potter 1971: 52–53 [comb. nov., provisionally recognized, Japan].

*Lethenteron matsubarai* Vladykov & Kott, 1978: 1792–1800 [original description, Shokotsu River, Hokkaido Island, Japan (44°22'N, 143°20'E)]; Vladykov and Kott 1979c: 10 [common name: Japanese brook lamprey; we recommend this common name now be used for *Le. mitsukurii*].

*Lampetra reissneri* (non Dybowski, 1869) – Sato 1984: 2 [dorsal and caudal fins without blackish parts in adults, Japan].

*Lethenteron kessleri* (non Anikin, 1905) – Iwata et al. 1985: 186–188 [in part, Hokkaido Island, Japan, individuals with unpigmented second dorsal and caudal fins].

*Petromyzon mitsukurii* – Paepke and Schmidt 1988: 160 [comb. nov., Japan].

### Synonymy of *Lethenteron kessleri* (Anikin, 1905), the Siberian brook lamprey

Possible synonym, new combinations, and misidentifications are included.

*Petromyzon kessleri* Anikin, 1905: 10–15 [original description, Tom’ River and at the mouth of its tributary the Kirgizka River, Ob’ River drainage, near Tomsk, Russia].

*Lampetra planeri* (non Bloch, 1784) – Berg 1906: 180–182 [in part, ZIN 6174, 6307, 6310, 6311, 7815].

*Lampetra planeri reissneri* (non Dybowki, 1869) – Berg 1911: 42–43 [in part, ZIN 6174, 6307, 6310, 6311, 7815].

Lampetra (Lampetra) japonica kessleri – Berg 1931: 93, 102–103 [comb. nov., Ob’ River drainage and Yenisei River, Siberia, Russia].

*Lampetra japonica kessleri* – Novikov 1966: 24–25 [Yakut common name: Bye-balyk, 134 mm TL female, July 1963, from Kolyma River, Russia]; Kuderskiy and Mel’nikova 1970: 16 [common name: Siberian lamprey, 139–173 mm TL spawning or spent adults, 13–16 June 1969, Yemtsa River, Severnaya Dvina River drainage, Russia]; Poltorykhina 1971: 281–285 [common name: Arctic brook lamprey, Bol’shoy and Malyy Krivoy channels of the Irtysh River, below Ust’-Kamenogorsk, Kazakhstan].

*Lampetra kessleri* – Poltorykhina 1974: 192–201 [common name: Siberian lamprey, side channel of the Irtysh River near Ust’-Kamenogorsk, Ul’ba and Cheremshanka rivers, upper Irtysh River system, Kazakhstan].

*Lampetra japonica* (non von Martens, 1868) – Savvaitova and Maksimov 1978: 556 [100–140 mm TL spawning individuals, 17–21 June 1972, Levyy Kolkalvayam River, tributary to the Utkholok River, western Kamchatka, Russia].

? *Lethenteron alaskense* Vladykov & Kott, 1978: 7–9, fig. 3 [original description, West Creek, a tributary to Brooks Lake, Naknek River system, Alaska, U.S.A.]; Vladykov and Kott 1979c: 10 [common name: Alaskan brook lamprey]; Sutton 2017: 1198 [Chena River, Yukon River drainage, Alaska, U.S.A.].

*Lethenteron kessleri* – Holčík 1986a: 197 [comb. nov.]; Holčík 1986b: 220–236; Sotnikov & Solov’ev 2002: 807–808 [common name: Siberian lamprey, 130–170 mm TL adults, 2 June 1993, Shipulovka River, Severnaya Dvina River drainage, Russia].

*Lethenteron camtschaticum* (non Tilesius, 1811) – Kucheryavyi et al. 2007a: 41–45, 47 [in part, Kolkavayam and Utkholok rivers, western Kamchatka, Russia, anadromous forma praecox, 145–220 mm mature individuals of both sexes combined, and resident form, 100–165 mm mature individuals of both sexes combined].

*Lethenteron reissneri* (non Dybowki, 1869) – Kottelat and Freyhof 2007: 43–44 [Severnaya Dvina and Pechora river drainages, Russia].

### Partial synonymy of *Lethenteron reissneri* (Dybowski, 1869), the Far Eastern brook lamprey based on this study

A more extensive synonymy is given in Renaud and Naseka (2015).

*Lampetra reissneri* – Mori 1936: 3, 6, 10, 15 [Maritime, Karafuto (= Sakhalin), Hokkaido, and Amur zoogeographical districts, Japan].

*Lethenteron japonica kessleri* (non Anikin, 1905) – Sato 1951: 58–59 [Shibechari River, now known as Shizunai River, Hokkaido Island, Japan, 26 spawning adults 144–193 mm TL].

*Lethenteron kessleri* (non Anikin, 1905) – Sato 1984: 2 [usually caudal fin but not dorsal fins with a blackish part in adults, Hokkaido Island, Japan]; Iwata et al. 1985: 186–188 [in part, Hokkaido Island, Japan, individuals with unpigmented dorsal fin, but pigmented caudal fin].

### Generic assignment of Lampetra (Eudontomyzon) morii

When Berg (1931) described this new non-migratory species from the upper Yalu River, in what was then known as Manchuria, but now more or less follows the border of the People’s Republic of China and North Korea, the only other members of the subgenus, *Lampetra danfordi* (Regan, 1911) and *L. mariae* Berg, 1931, were European in distribution. To explain the discontinuous distribution of the subgenus, he suggested two hypotheses: 1) the European and Asian species were relics of a former continuous distribution or 2) they arose independently, the former from *La. fluviatilis* and the latter from *La. japonica* (= *Le. camtschaticum*). However, in their cladistic study based on morphological characters Gill et al. (2003) showed that *Eudontomyzon danfordi* and *E. morii* constituted a clade supported by a single synapomorphy, the presence of alate rows of teeth in the laterals fields, and that this clade was sister to *La. fluviatilis*–*L. ayresii* rather than *Le. camtschaticum*. The four specimens of *E. morii* used in Gill (2003) included a syntype and three non-type adults from the upper Yalu River, People’s Republic of China.

Later studies based on the mitochondrial DNA (Lang et al. 2009; White 2014; Pu et al. 2016; Ren et al. 2016) have suggested that *E. morii* should be assigned to the genus *Lethenteron*. However, the material used in these studies is of unconfirmed identity. In Lang et al. (2009) and White (2014) the specimen was a metamorphosing individual with exolaterals, but from the Liaohe River, west of the Yalu River. Although Berg (1931) suggested that *La. morii* probably occurred in the Liao-ho (= Liaohe), he had only seen an ammocoete from that river. Additionally, exolaterals have occasionally been reported in *Lethenteron* species (see above ‘*Lethenteron* species with exolaterals’, page 23). The specimen was the same in Pu et al. (2016) and Ren et al. (2016) and came from the city of Dandong, at the Yalu River estuary, Yellow Sea basin, and may in fact have been *Le. camtschaticum*. Unfortunately, no morphological description of the specimen is provided. Given the uncertainty surrounding the identity of the specimens used in these molecular studies, we prefer to continue treating *E. morii* in the genus *Eudontomyzon*. According to Renaud (1982), who examined four adults from the type locality, including a syntype, *E. morii* possesses one or two exolateral rows on either side of the oral disc (Fig. 6) and this distinguishes it from members of the genus *Lethenteron* that usually have no exolateral teeth, rarely one or two teeth and exceptionally one row.

### Taxonomic key to adults of *Lethenteron*

*Lethenteron* adults are characterized by the presence of two dorsal fins; spade-like caudal fin; supraoral lamina with a wide bridge bearing a unicuspid (rarely bicuspid) tooth at each end and rarely one or two unicuspid teeth on the bridge; three (rarely four) endolateral bicuspid teeth on either side of the oral disc (rarely the third tooth may be unicuspid or tricuspid and the fourth tooth unicuspid); infraoral lamina usually with lateralmost bicuspid teeth and unicuspid teeth internally, but much variability exists within the genus (see Table 10); one or two (usually one) rows of posterial teeth; no exolateral teeth (rarely one or two teeth on one or both sides, and exceptionally, a complete row on both sides); transverse lingual lamina with an enlarged median cusp. The key was constructed based on the character matrix compiled in Table 10.

**Table 10.** Character matrix of species of *Lethenteron*. Abbreviations: b, bicuspid; u, unicuspid. Pigmentation coverage as follows: -, absent to < 1%; +, 1% to < 25%; ++, 25% to < 75%; +++, ≥ 75%. Percentages in parentheses are percentages of occurrence of character states.

Species	Trunk Myomeres	Infraoral Lamina	Transverse Lingual Lamina	Pigmentation		
				Gular	Second Dorsal Fin	Caudal Fin
*Le. alaskense*	66–72^1^	6–11 teeth (the lateralmost and sometimes an internal one bicuspid; the others unicuspid)^1^	9–15 teeth, the median one enlarged^1^	-^1^	with blotch (94%)^1^, no blotch (6%)^1^	+(17%)^1^, ++(37%)^1^, +++(46%)^1^
*Le. appendix*	64–70^2^, 66–74^3^	6–10 teeth (the lateralmost unicuspid or bicuspid; the others unicuspid)^2,3^	9–15 teeth, the median one enlarged^2,3^	+++^3^	with blotch (38%)^3^, no blotch (62%)^3^	+(44%)^3^, ++(26%)^3^, +++(30%)^3^
*Le. camtschaticum*	65–73^4^, 70–75^5^, 72–77^6^	1b4u1b^5^, 1b5u^5^, 6u1b^6^	6u–I–2u^5^, 3u–I–2u^5^, 4u–I–4u^6^	-^4^	with blotch (100%)^4, 15^	+(27%)^4^, ++(53%)^4^, +++(20%)^4, 18^
*Le. kessleri*	70–74^7^	1b4u1b^7^, 1b5u1b^7^	5u–I–7u^7^, 7u–I–7u^7^	-^12^, +^12^	with blotch (100%)^16^	+++(100%)^19^
*Le. mitsukurii*	66–70^8^	6u^8^	2u–I–4u^10^, 3u–I–3u^10^	-^13^	no blotch (100%)^17^	-(100%)^17^
*Le. ninae*	58–62^9^	7u^9^, 1b3u1b^9^, 5u1b^9^, 1b5u^9^, 6u1b^9^, 1b4u1b^9^, 3u1b3u^9^, 1b1u1b1u1b^9^	6u–I–5u^9^, 4u–I–4u^9^, 4u–I–5u^9^, 5u–I–5u^9^, 5u–I–6u^9^, 7u–I–7u^9^, 2u–I–2u^9^, 3u–I–2u^9^, 4u–I–3u^9^, 5u–I–4u^9^, 7u–I–5u^9^, 8u–I–7u^9^	-^14^	with blotch (100%)^9^	+(60%)^20^, ++(33%)^20^, +++(7%)^20^
*Le. reissneri*	70–72^6^	1b5u^6^, 1b4u1b^6^	2u–I–2u^11^	-^11^	no blotch (100%)^11^	+++(100%)^11^

^1^Vladykov and Kott (1978a). ^2^Vladykov (1949) as *Entosphenus lamottenii* [Robins et al. (1980) state that *Petromyzon lamottenii* Lesueur, 1827 is unidentifiable and use the next available name *Lampetra appendix* (= *Lethenteron appendix*) for the American brook lamprey]. ^3^Vladykov and Kott (1978a as *Lethenteron lamottenii*, see note no. 2). ^4^Vladykov and Kott (1978a as *Lethenteron japonicum*). ^5^ this study, Table 2 (based on type material of *Petromyzon marinus camtschaticus* and *Petromyzon japonicus*). ^6^Renaud and Naseka (2015). ^7^ this study, Table 2 (based on type material of *Petromyzon kessleri*). ^8^ this study, Table 8 (based on type material of *Lampetra mitsukurii* and synonym *Lethenteron matsubarai*). ^9^Naseka et al. (2009), Tuniyev et al. (2016). ^10^ this study, Table 8 (based on type material of *Lethenteron matsubarai*, synonym of *Lampetra mitsukurii*). ^11^Renaud and Naseka 2015 (based on specimen from Shangshi River). ^12^ this study, Table 6 (based on three specimens from the Ob’ River identified by Berg (1931) as *Lampetra japonica kessleri*). ^13^ this study, Table 9 (based on type material of *Lethenteron matsubarai*, synonym of *Lampetra mitsukurii*). ^14^ this study (based on CMNFI 2008–59, paratype of *Lethenteron ninae*). ^15^ this study, Table 3 (based on type material of *Petromyzon marinus camtschaticus* and *Petromyzon japonicus*); Tilesius 1811. ^16^Anikin (1905). ^17^ this study, Table 9 (based on type material of *Lampetra mitsukurii* and synonym *Lethenteron matsubarai*). ^18^ this study, Table 3 (based on type material of *Petromyzon marinus camtschaticus* and *Petromyzon japonicus*). ^19^ this study, Table 6 (based on one specimen from the Ob’ River identified by Berg (1931) as *Lampetra japonica kessleri*). ^20^Tuniyev et al. (2016).

**Table:** 

1	Caudal fin pigmentation absent to < 1% coverage (i.e., -) [Japan]	***Le. mitsukurii***
–	Caudal fin pigmentation between 1% and 100% coverage (i.e., +, ++, +++)	**2**
2	Trunk myomeres 58–62 [western Transcaucasia]	***Le. ninae***
–	Trunk myomeres > 63	**3**
3	Gular pigmentation between 75 and 100% coverage (i.e., +++) [eastern North America]	***Le. appendix***
–	Gular pigmentation absent to < 25% coverage (i.e., -, +)	**4**
4	Second dorsal fin unpigmented (no blotch at the apex) [Asia]	***Le. reissneri***
–	Second dorsal fin pigmented (blotch at the apex)	**5**
5	Parasitic mode of life; individuals reaching up to 790^1^ mm TL [Eurasia and North America]	***Le. camtschaticum***
–	Nonparasitic mode of life; individuals reaching ≤ 230 mm TL [Eurasia and western North America]	***Le. kessleri* , *Le. alaskense***

### Phylogenetic considerations

The three phylogenetic studies that examined the most comprehensive sets of *Lethenteron* species (Yamazaki et al. 2006; Lang et al. 2009; White 2014) did not achieve any resolution among the species treated, and therefore their relationships cannot be established. The strict consensus tree generated from maximum parsimony in Yamazaki et al. (2006) was based on the partial mtDNA COI gene (1,009 bp) and that in Lang et al. (2009) on the partial mtDNA cyt b gene (1,133 bp). The tree generated from maximum-likelihood in White (2014) was based on the mtDNA ND3 gene (351 bp) and a portion of the mitochondrial control non-coding region I (340 bp). Additionally, Artamonova et al. (2015) examined nucleotide sequence diversity of the partial mtDNA COI gene (1,072 bp) in Eurasian *Lethenteron* using median joining network analysis and concluded that the heterogeneity in the distribution of the haplotypes does not give any grounds to assume that speciation has occurred. The four studies included material identified as *Le. camtschaticum*, *Le. reissneri* and/or *Le. kessleri*. Additionally, Lang et al. (2009) and White (2014) included *Le. alaskense* and *Le. appendix*. None of the studies contained material identified as *Le. mitsukurii* or *Le. ninae*. According to Yamazaki and Goto (2016), the reason we do not see genetic differences between *Le. camtschaticum* and nonparasitic forms derived from it, is that the latter are the result of phenotypic plasticity rather than genetic polymorphism. However, Docker and Potter (2019) state that the lack of fixed differences in mtDNA sequences is not in itself evidence of phenotypic plasticity nor is demonstration of fixed genetic differences evidence of species-level differences. We do not treat *Lethenteron* sp. N and *Le. * sp. S here because neither have been formally described. We recognize that taxonomic changes will need to be made once they are. In order to achieve better resolution among the *Lethenteron* species that have been formally described, we suggest that a total evidence cladistic analysis which includes both morphological and molecular characters, including nuclear genes, be performed.

## Footnotes

Orlov et al. (2014).
